# The Effects of TLR Activation on T-Cell Development and Differentiation

**DOI:** 10.1155/2012/836485

**Published:** 2012-06-07

**Authors:** Bo Jin, Tao Sun, Xiao-Hong Yu, Ying-Xiang Yang, Anthony E. T. Yeo

**Affiliations:** ^1^Department of Gastroenterology, The 309th Hospital of The People's Liberation Army, Beijing 100091, China; ^2^Department of Infectious Diseases, Naval General Hospital, Beijing 100048, China; ^3^Savient Pharmaceuticals, East Brunswick, NJ 08116, USA

## Abstract

Invading pathogens have unique molecular signatures that are recognized by Toll-like receptors (TLRs) resulting in either activation of antigen-presenting cells (APCs) and/or costimulation of T cells inducing both innate and adaptive immunity. TLRs are also involved in T-cell development and can reprogram Treg cells to become helper cells. T cells consist of various subsets, that is, Th1, Th2, Th17, T follicular helper (Tfh), cytotoxic T lymphocytes (CTLs), regulatory T cells (Treg) and these originate from thymic progenitor thymocytes. T-cell receptor (TCR) activation in distinct T-cell subsets with different TLRs results in differing outcomes, for example, activation of TLR4 expressed in T cells promotes suppressive function of regulatory T cells (Treg), while activation of TLR6 expressed in T cells abrogates Treg function. The current state of knowledge of regarding TLR-mediated T-cell development and differentiation is reviewed.

## 1. Introduction

Innate immunity protects the host from pathogenic infectious agents. Every infectious microorganism possesses conserved molecular structures, for example, lipopolysaccharide, peptidoglycan, flagellin, microbial nucleic acids and these are collectively referred to as pathogen-associated molecular patterns (PAMPs) [1]. PAMPs are recognized by corresponding germline-encoded pattern recognition receptor (PRR) expressed on innate immune cells of the host, for example, dendritic cells (DCs), macrophages and neutrophils [2, 3]. This triggers various signal pathways to produce inflammatory responses and adaptive immunity [4, 5].

At least 5 classes of PRRs have been characterized: Toll-like receptors (TLRs), retinoic-acid-inducible gene-I- (RIG-I-) like receptors (RLRs), nucleotide-binding domain and leucine-rich repeat containing gene family (alternatively named NOD-like receptors, NLRs), C-type lectin receptors (CLRs) and cytosolic DNA receptors (CDRs) [4, 6]. TLRs are membrane-bound receptors that sense PAMPs on the cell surface or in endosomes [7], while RLRs and NLRs recognize microbial molecules in the host cytosol [8]. CLRs are primarily expressed in myeloid cells and recognize polysaccharide structures of pathogens inducing immune responses [6, 9]. With the exception of TLR9, CDRs are a new family composed of at least 6 members that also trigger innate immunity upon detecting cytosolic DNA [10, 11]. TLRs were initially discovered in 1997 [12] and represent a canonical family of PRRs that govern adaptive immune response by inducing a Th1-skewed response, immunoglobulin G2c production and antigen-specific cytotoxic T lymphocyte (CTL) response [13–15].

**Figure 1:**
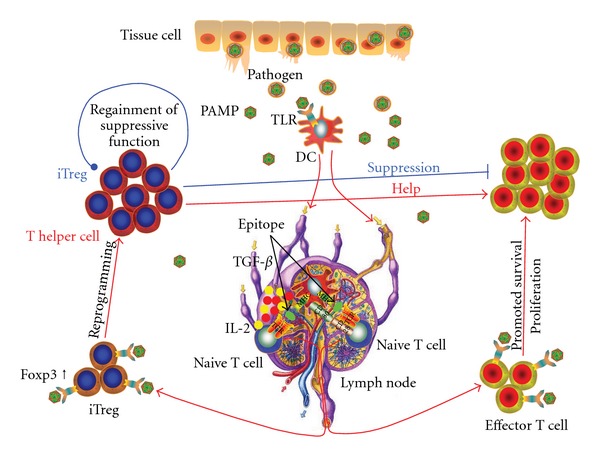
The effects of TLR on T-cell activation. PAMPs from invading pathogens bind with TLRs expressed in DCs, which causes DC activation. Activated DCs migrate to the draining lymph nodes where, in the presence of co-stimulatory signals and instructing cytokines, they present the antigen epitope with MHC molecules to activate naive T cells. DCs also induce iTreg in the presence of TGF-*β* and IL-2. These activated T cells move to the site of infection to fight against the invading pathogen. Activation of TLRs in activated T cells induces their survival and clonal expansion. Direct engagement of TLR in iTreg cells promotes their expansion with reduced suppressive function and reprograms them to differentiate into T helper cells, which in turn provide help to effector cells. When the infected pathogen is eliminated, the clearance of TLR ligands results in the suppressive function of the expanded iTreg cells being restored. This serves to regulate the expanded effector T-cell population.

Upon recognition of foreign antigen for DCs via the TLR-PAMP interaction [4, 16], immature DCs resident in tissues mature into professional antigen-presenting cells (APCs) to induce effector and memory T-cell responses in lymphoid organs. Additionally, DCs are capable of inducing antigen-specific T-cell tolerance immunosuppression (Figure 1) [16]. T cells are divided into different subsets based on their phenotypes, intracellular molecules expression, cytokine production, the lengths of telomeres and state of immunity [17]. The current knowledge of TLRs activation in relation to T-cell activation and differentiation is presented here.

## 2. T Lymphocyte Development and Subsets Differentiation

### 2.1. T-Cell Development in Thymus (Figure 2)

Thymic T-cell progenitors are believed to come from circulating hematopoietic stem cells originating from bone marrow. All peripheral T cells are developed from these progenitor cells [18–20]. The entry of T-lymphoid progenitor cells at an early embryonic developmental stage before vascularization of thymus, or at later embryonic and postnatal stages after vascularization, initiates development of T cells in the thymus [21, 22]. Thus, T progenitor cells can travel to and reside in thymus via either a nonvascular route at an early embryonic developmental stage or via a vascular way at late embryonic and postnatal stages. Chemokines such as C-C chemokine receptor type 7 (CCR7) and CCR9 play a role in the prevascular colonization of T-cell progenitors into the thymus primordium [23], while the combination of P-selectin and P-selectin glycoprotein ligand-1 is involved in postnatal thymus seeding [22]. These cells initially express neither CD4 nor CD8 and are referred to CD4/CD8 double-negative (DN) thymocytes [24]. Such DN thymocytes migrate from the corticomedullary junction to the subcapsular region of the cortex and sequentially transform into DN1 (CD44^+^CD25^−^), DN2 (CD44^+^CD25^+^), DN3 (CD44^−^CD25^+^) and DN4 (CD44^−^CD25^−^) [25–27] cells with weak expression of CD4, CD8, CD25 and CD44. These are the direct precursors of CD4/CD8 double-positive (DP) thymocytes [28]. DP thymocytes develop in thymus cortex from pre-DP where son of sevenless gene 1 (Sos1), a guanine nucleotide exchange factor for Ras, plays a pivotal role during this transition [29]. DP thymocytes express TCR*αβ* on the cell surface and these interact with self-peptide-MHC complexes presented by cortical thymic epithelial cells (cTECs) for positive selection (i.e., survival) or negative selection (clonal deletion, i.e. death). The process is determined by avidity and aggregation of TCR with the ligand interacting with one another [30]. Development of single positive (SP) lineages of CD4^+^CD8^−^ or CD4^−^CD8^+^ thymocytes is determined during positive selection [20] and the properties of protein degradation and self-peptide presentation of cTEC may play a role in SP lineages positive selection [30, 31].

Positively selected thymocytes migrate to the medulla via CCR7-mediated chemotaxis [30]. The medullary TECs (mTEC) ectopically express multifarious “tissue-specific” antigens (TSAs)/peripheral tissue-restricted antigens (PTAs), that is, promiscuous gene expression representing peripheral tissues [32, 33]. This expression is partially controlled by the transcription factor autoimmune regulator (AIRE) [34]. Antigens from either apoptotic mature mTECs or peripheral tissues are taken up by thymic DCs and cross-presented to developing thymocytes to induce negative selection of self-reactive thymocytes establishing self-tolerance [30]. It is suggested that circulating DCs bearing peripheral tissue antigens are also recruited intrathymically for cross-presentation and therefore involved in clonal deletion [35]. Mature thymocytes that have completed T-cell development emigrate from thymus through perivascular space in the corticomedullary junction and medulla [36] to peripheral lymphoid organs. T-cell emigration is regulated by sphingosine-1-phosphate receptor 1 [37, 38]. Different subsets of T cells may have different affinities for blood/lymphatic vessels and these determine the routes of emigration [32]. A new subset phenotypically and functionally distinct from peripheral naive T cell that emigrates from the thymus referred to recent thymic emigrants (RTEs) requires further maturation in secondary lymphoid organ to become functionally competent peripheral T cells [39].

Self-tolerance is induced in thymus either by negative selection or by natural regulatory T cells (nTreg) development. Most of the nTreg cells are derived from CD4^+^ SP thymocytes residing in the medullary compartment of the thymus [40, 41]. It is hypothesized that tolerance of uncommon self-antigens such as myosin usually presents after muscle injury is preferentially recognized by TCR and mediated by nTreg cells. By contrast, cells that are involved in chronic engagement of TCR/CD28 signaling by recognizing ubiquitous antigen, for example, albumin, the 5th component of complement, insulin, are negatively selected [40, 42, 43]. Decreased presentation of cognate antigens on mTECs or DCs can induce nTreg cell development [44]. Distinct APC subsets may preserve different TCR specificities and their ability to mediate negative selection [40, 45–47]. It has been suggested that forkhead box P3 (Foxp3) negative nTreg cell precursors, induced by TCR signaling, can use interleukin-2 (IL-2), IL-15, or IL-7 to activate Foxp3 expression without the need for additional TCR signals [40]. It is believed that nTreg cell development begins early at the DP stage in pediatric thymus. Foxp3^+^ DP thymocytes with a functional IL-7 receptor and upregulated expression of Bcl-2 protect themselves from being negative selected. Foxp3^+^ DP thymocytes that express CD103 are possible precursor of Foxp3^+^ CD8^+^ SP cells [48]. Hassall's corpuscles, groups of epithelial cells in the thymic medulla, may serve as specialized small niches required to support nascent nTreg cell development [49].

### 2.2. Development of T-Cell Peripheral Tolerance (Figure 3)

In addition to the tolerance induced in thymus, autoreactive T cells that have escaped from negative selection in thymus due to low avidity of TCR to self-peptide-MHC complex [50] or insufficiently expressed TSA in mTECs will be deleted (cell death) or inactivated (anergy) in periphery, that is, peripheral tolerance [42].

Lymph nodes are a primary location where peripheral tolerance takes place. It has been demonstrated that lymph node stromal cells (LNSCs), similar to mTECs in thymus, are able to express a variety of TSAs to induce immune tolerance of T cells [51]. The peripheral expression of TSAs is either AIRE dependent [52, 53] or independent [54]. Another regulating factor, deformed epidermal autoregulatory factor 1 (Deaf1) is also involved in PTAs expression [55]. Deaf1 variant isoforms inhibit the transcriptional activity of canonical Deaf1 and this suppresses PTA expression [55]. Deaf1 transcript has been detected in every subset of LNSCs [56]. All the subsets of LNSCs can express TSAs and present TSAs to activate antigen-specific CD8^+^ T cells under both steady-state and inflammatory conditions [56]. By contrast, cross-presentation of TSAs produced from LNSCs by lymph node resident DCs does not seem to play an important role [57]. Although TSA proteins expressed by LNSCs might be functional [51, 58], the expression of TSA protein by LNSCs is different from its expression in peripheral tissue. This is evidenced by the fact that even the products from a single type of differentiated peripheral cell can be produced separately from distinct subsets of LNSCs, for example, both the protein of mlana gene expression and tyrosinase are products of terminally differentiated melanocytes, their mRNA expression as PTAs in lymph node is segregated in fibroblastic reticular cells (FRCs) [56] and lymphatic endothelial cells (LECs) [54], respectively. In addition to TSAs expressed by LNSCs, lymph can also serve as a source of self-antigens to induce peripheral tolerance in lymph node [59]. Compared with plasma, lymph contains more processed protein fragments and peptides from draining organs or tissues [60] and thus a significant pool of self-antigen for the induction of peripheral tolerance [59].

LNSCs are reported capable of upregulating co-stimulatory molecules to induce T-cell lineage deletion rather than activation [56]. The role of LNSCs in the induction of Treg cell is unknown [51]. It has been suggested that autoimmunity is promoted by induction of self-antigen specific effector-memory T cells when their TCR is continuously engaged at sites of high TSA expression under conditions of tissue injury, infection and/or inflammation [42]. Without inflammation, DCs resident in peripheral lymph organs would induce tolerance in naive T cells bearing TCR with high avidity for self-antigen and incomplete maturation of DC also serves tolerance induction [42]. The peripheral deletion of autoreactive T-cell lineage is mediated by an apoptosis involving activation of Fas receptor by Fas ligand and inactivation of survival protein B cell lymphoma 2 (Bcl-2) by its antagonist Bcl-2-interacting mediator of cell death (Bim) [42, 61]. A nonapoptotic mechanism of peripheral deletion was recently identified in which autoreactive CD8^+^ T cells actively invade hepatocytes in liver and are degraded in the endosome/lysosome of the hepatocytes [62]. This process is known as emperipolesis [63] and has been described as early as the 1920s [64]. The invasion of T cells into hepatocyte is dependent on T-cell activation, filamentous actin reorganization, myosin light chain kinase, as well as other kinases like PI3K. Inhibition of this suicide emperipolesis by wortmannin, a kinase inhibitor capable of inhibiting T-cell invasion into hepatocytes *in vivo*, is associated with accumulation of autoreactive CD8^+^ T cells in the liver, and breach of tolerance results in the development of autoimmune hepatitis [62]. By interrupting costimulation, functional tolerance of T cell, that is, anergy can be developed and maintained by counter-regulatory receptors such as cytotoxic T lymphocyte-associated antigen-4 (CTLA-4) that shares structural similarity with CD28 capable of binding CD80 and CD86 and blocking CD28 costimulation [42, 65]. Another counter-regulatory molecule, programmed cell death-1 (PD-1) is also crucial for the maintenance of peripheral tolerance [65].

### 2.3. Development of Mucosal Tolerance

Mucosa discussed here are those that line the gastrointestinal system and the respiratory system including nasal passages. The largest immune organ of the body is the gut-associated lymphoid tissue (GALT) consisting of Peyer's patches and isolated lymphoid follicles [66] located within the small intestine. Each meter of human intestine has approximately 10^12^ lymphoid cells [67]. GALT processes dietary antigens and is responsible for immunotolerance toward intestinal commensal flora.

Intestinal commensal microbiota is essential for adaptive and innate immunity. In germ-free mice, the absence of these bacteria results in impaired local and systemic immune responses. This is evidenced by a reduced number and smaller sized Peyer's patches, a reduced number of mesenteric lymph nodes and diminished IgA and IgG production [66, 68–70].

Metabolites of intestinal microbiota, for example, in mice with dextran-sulfate-sodium- (DSS-) induced colitis, short-chain fatty acids such as acetate, a fermented product of *Bifidobacterium* when it acts on dietary fiber, interact with G-protein-coupled receptor 43 and stop the differentiation of IL-17-producing cells in the lamina propria [71]. Metabolites from food and food proteins also determine susceptibility to systemic infection, immunoreactivity and immune tolerance [72–75]. A unique property of mucosa when exposed to ingested antigens is suppression of immune responses to subsequent parenteral challenges with the same antigen [76, 77]. This physiologically induced tolerance is referred to as oral tolerance [66, 67, 78, 79]. Mucosal DCs can produce TGF-*β*, IL-10 and induce CD103^+^ DCs to promote Tregs induction [80, 81]. Resident lamina propria CD103^+^ DCs can promote Foxp3^+^ Treg cell differentiation and induce gut-homing receptors, for example, CCR9 and *α*4*β*7 integrin expression in T cells [82].

The orally ingested antigen can be taken up by a variety of mechanisms. Microfold cells (M cells) are specialized epithelial cells without microvilli and thick glycocalyx in the small intestine overlying Peyer's patches and lymphoid follicles and are responsible for transcytosis [69]. These cells express TLR4, platelet-activating factor receptor, *α*5*β*1 integrin and galectin-9 on cell surfaces that enable M cells to sense and transport intestinal antigens into intraepithelial pockets to be processed by APCs [83]. Intestinal columnar epithelial cells are also capable of transporting luminal antigens through these PRRs [83] or the epithelial-associated neonatal Fc receptor to secrete and combine IgG or IgG-antigen complexes to cross mucosal epithelial cells [84]. DCs by their cellular processes which traverse the epithelium without disrupting tight junctions can sense luminal antigens [85, 86].

A variety of regulatory mechanisms are involved in oral tolerance. The amount of ingested antigen is a major factor that determines the mechanism of oral tolerance. Generally, low amounts of antigen result in Treg induction while higher doses lead to immune cell anergy or clonal deletion [67]. Activation of mesenteric lymph node CD103^+^ DCs preferentially induces Foxp3^+^ Treg cells differentiation from Foxp3^−^ naive conventional CD4^+^ T cells in the presence of TGF-*β* and the dietary vitamin A metabolite, retinoic acid [81, 87]. CD103^+^ DCs express a retinal dehydrogenase, aldehyde dehydrogenase family 1 subfamily A2 that can convert retinal or vitamin A into retinoic acid. This facilitates Foxp3^+^ iTreg cell induction [81]. Even in the absence of thymus-derived nTregs, the development of antigen-specific CD4^+^CD25^+^Foxp3^+^CD45RB^low^ cells that are anergic and suppressive can occur [76]. Gut CD103^+^ DCs also expresses indoleamine 2, 3-dioxygenase (IDO) involved in the activation of Foxp3^+^ iTreg cells and hence oral tolerance [88]. TGF-*β* can transform IDO^−^ DCs into IDO^+^ DCs in mice and prostaglandin E2 plays similar role in human [67]. This process involves intracellular signaling for the self-amplification and maintenance of a stable regulatory phenotype in pDCs [89].

All major types of regulatory T cells are involved in oral tolerance, including thymic-derived nTreg, mucosally induced iTreg, IL-10 secreting CD4^+^CD25^low^CD45RB^low^ type 1 regulatory T cell (Tr1 cell), TGF-*β*-dependent latency-associated peptide (LAP)^+^ Th3 type Treg and CD8^+^ Treg [67]. LAP is a propeptide capable of combining TGF-*β* to constitute a latent TGF-*β* complex [90]. It has been suggested that after exposure of oral antigen, CD4^+^CD25^−^Foxp3^−^LAP^+^ Th3 cells produce TGF-*β* to support CD4^+^CD25^+^Foxp3^+^ nTreg cells, induce CD4^+^Foxp3^−^ T-cells differentiation into Foxp3^+^CD25^+^LAP^−^iTreg cells and suppress Th1 and Th2 responses [67]. iTreg cells may modulate DCs to produce IL-27 which induces IL-10-producing Tr1 cells [91]. Foxp3^+^ iTreg cells are essential for mucosal tolerance development [92]. Oral tolerance can also be elicited by oral administration of anti-CD3 monoclonal antibody instead of application of cognate antigen to activate TCR and induce Th3 type CD4^+^CD25^−^LAP^+^ Tregs in mesenteric lymph nodes [93]. Oral exposure to ligands of aryl hydrocarbon receptor is also capable of inducing Foxp3^+^ Treg and Tr1 cells by acting on both T cells and DCs producing IL-27, retinoic acid and IL-10 in the gut [94, 95].

Nasal administration of antigen preferentially induces IL-10-dependent Treg cell development, for example, Tr1 cell and CD4^+^CD25^−^LAP^+^ Treg cell [67, 96, 97]. As the antigen exposed to respiratory mucosa does not exert digestion that occurred in the gut, the antigen dosage required to induce nasal tolerance is smaller than that needed in the induction of oral tolerance [98]. DCs that produce IL-10 in the lungs are critical in the induction of IL-10-secreting Tr1 cell development which elicits nasal tolerance [99]. The CD4^+^Foxp3^+^ Treg cells expressing membrane-bound TGF-*β* also participates in nasal tolerance [100]. CCR7-dependent migration of CD103^+^ and CD103^−^ pulmonary dendritic cells to the bronchial lymph node is indispensable for nasal tolerance induction [101]. CD11b^+^ and CD103^+^ DCs are the major DC subsets in the lung. In contrast to the actions in the gut, pulmonary CD103^+^ DCs appears to prime Th2 responses to the inhaled antigen while CD11b^hi^ DCs elicit Th1 responses [102].

### 2.4. T-Cell Subsets Development and Differentiation in Periphery (Figure 2)

CD4^+^ T cells play critical roles in the functioning of the host immune system. Upon stimulation, peripheral CD4^+^ T cells can differentiate into T helper (Th) cells or inducible Treg cells (iTreg). Currently, at least 4 Th cell subsets have been identified, Th1, Th2, Th17 and iTreg [103]. T follicular helper (Tfh) has been suggested as a new subset of Th family [104–106]. There is debate whether new subsets such as Th9, Th22 [107–109] are separate lineages [103, 110].

APCs take up antigen and digest it in the cytosol to process the epitope. The epitope is then presented together with MHC molecules to TCR on the T-cell surface. Simultaneously, APCs also secrete co-stimulatory molecules for example, CD80, CD86 that bind the co-stimulatory receptor of T cells, for example, CD28. Thus all 3 elements are required for T-cell activation, that is, epitope, MHC molecules and costimulation signals. Upon TCR activation, T cells produce CD154 (alternatively named CD40L) to bind CD40 on the cell surface of APCs to further activate APCs. The lineage commitment of Th cells is determined by the cytokine milieu, transcription factors and co-stimulatory molecules such as CD28, CD154. The transcription factors involved in this process are activated by TCR signaling [16, 103]. IL-12 [111] and interferon (IFN) *γ* [112] are essential for the induction of the Th1 cells. When cognate antigen stimulation is present, IL-4 and IL-2 are required by the naive CD4^+^ T cells to differentiate into IL-4-producing Th2 cells [113, 114]. Transforming growth factor- (TGF-) *β* stimulates naive CD4^+^ T cells either to differentiate into Th17 cells in the presence of IL-6 or alternatively differentiate into iTreg cells in the presence of IL-2 (or IL-1*β* in human) [115–117]. In the absence of IL-6 and in the presence of TGF-*β* and IL-21, Th17 cells can also be induced [118]. Primed CD4^+^ T cells are also able to differentiate into Tfh cells in the presence of IL-6 (mice) or IL-12 (human) expressing IL-21 [119–122]. IL-21 can promote Tfh cell differentiation by feedback. Therefore, it has been proposed that major products of the differentiated cells, for example, IFN-*γ* from Th1, IL-4 from Th2, IL-17 from Th17, IL-21 from Tfh, play critical roles in its self-induction [103].

Newly primed CD4^+^ T cells are programmed by various cytokines and other factors from DCs to produce transcription factors. T box expressed in T cells (T-bet) is a major factor for Th1 cell differentiation and IFN-*γ* production [123]. It can induce chromatin remodeling of IFN-*γ* alleles and IL-12 receptor (IL-12R) *β*2 expression and this promotes IFN-*γ* production as well as Th1 cell expansion induced by IL-12 [124]. However, in mature Th1 cells, reiteration of IFN-*γ* expression and stable chromatin remodeling are relatively independent of T-bet activity [125]. Signal transducer and activator of transcription (STAT) protein 4 and STAT1 are involved in Th1 cell differentiation. STAT4 is activated by IL-12 leading to Th1 and Th17 cells differentiation. IFN-*γ* production also occurs with nuclear factor *κ*B (NF-*κ*B) with multiple cis elements being involved [126, 127]. STAT1 can be activated by IFN-*γ* and serves as a regulator of T-bet activation and subsequent IL-12R expression *in vitro* [128]. The role of IFN-*γ*/STAT1 autocrine pathway in CD4^+^ T-cell differentiation *in vivo* is not fully understood [103].

GATA3, a member of GATA transcription factor family capable of binding to the DNA sequence “GATA,” is the master regulator of Th2 [129]. Without GATA3, Th2 cell differentiation is completely abolished both *in vivo* and *in vitro* [130, 131]. GATA3 can bind to 1279 genes in Th2 cells and 17 genes in 26 highly Th2-specific STAT6-dependent inducible genes. Among the 26 Th2-specific genes, 10 showed GATA3-dependent transcription while the remaining 16 genes were STAT6 dependent [132]. Production of Th2 cytokines is also promoted by GATA3 binding to promoters of IL-5 [133], IL-13 [134], and enhancers of IL-4 [135]. GATA3 has the ability to instruct Th2 commitment, promote Th2 cell expansion, suppress Th1 cell differentiation, thus facilitating Th2 differentiation [103].

STAT6 and STAT5 are essential in Th2 cell differentiation and expansion [136–139]. *In vitro* studies showed that activation of STAT6 is necessary and sufficient for Th2 cell differentiation with expansion triggered by IL-4 [140]. However, Th2 lineage commitment can still be induced by activation of GATA3 in a STAT6-independent manner *in vivo* [141]. Thus, it is possible that other transcription factors beside STAT6 may be involved in GATA3 activation. A recent report suggested that T-cell factor 1 (TCF-1) participated in GATA3 activation and promoted STAT6-independent IL-4-producing Th2 cell differentiation [142]. However, TCF-1 expression can be suppressed by IL-4 mediated by STAT6. Thus, the fine-tuning mechanism of Th cell polarization has a multichannel pattern [143]. STAT6 is also involved in the expression of Th2-specific cytokines, for example, IL-24 is mediated by the coordinate action of STAT6 and c-Jun transcription factors at the transcriptional level [144]. Recently, it was reported that STAT3 cooperates with STAT6 in promoting Th2 cell development [145]. A strong STAT5 signaling, correlated with higher expression of CD25, is required for Th2 and iTreg cell differentiation. By contrast, weak STAT5 signaling causes cell proliferation and survival of Th1 and Th17 cells [103]. *In vivo*, promiscuous expression of an activated form of STAT5 suppresses the production of both Th1 and Th17 cytokines and promotes the development of Th2 lineage cells [137].

The master regulator of Th17 cell is retinoic acid receptor related orphan receptor-*γ*t (ROR-*γ*t) [146, 147]. ROR-*γ*t deficiency results in significant reduction in IL-17 production. The residual IL-17 production in ROR-*γ*t-deficient cells appears to be attributed to ROR-*α*. Dual deficiency of ROR-*γ*t and ROR-*α* completely abolished IL-17 production [147]. SR1001, a high-affinity synthetic ligand binding to the ligand-binding domains of both ROR-*γ*t and ROR-*α* that induces a conformational change within the ligand-binding domain, is capable of reducing affinity for coactivators and increasing affinity for corepressors. This results in suppression of the receptors' transcriptional activity. Blocking the activities of ROR-*γ*t and ROR-*α* with SR1001 can inhibit Th17 cell differentiation and function and suppress cytokines expression in mature Th17 cells [148]. STAT3 is involved in Th17 cell differentiation, expansion and maintenance [103, 149]. Stimulation of the common precursor cell of Treg/Th17 by IL-6 activates STAT3 signaling and induces IL-21 expression [150]. IL-21 induces Th17 differentiation, suppresses Foxp3 expression and maintains a sustained STAT3 activation in a self-service autocrine pattern, that is, Th17 cells secrete IL-21, which in turn causes Th17 cells to induce cell differentiation [151]. STAT3 can also be activated by IL-23 and is responsible for the induction of ROR-*γ*t and IL-23R allowing the persistence of Th17 cells [103, 150, 152].

Treg cell development is controlled by the transcription factor Foxp3 [153, 154]. Mutation of Foxp3 gene results in fatal autoimmune disorders in human, for example, immune dysregulation, polyendocrinopathy, enteropathy, X-linked (IPEX) syndrome or in mice, for example, lymphoproliferative disorder and stable expression of Foxp3 is essential for immune homeostasis [155, 156]. Foxp3 is required for Treg lineage commitment, differentiation, expansion and function [153, 154, 157]. Sustained expression of Foxp3 in the mature Treg cell is essential to maintain the existing phenotype status and to execute the immunosuppressive function of Treg cell. Reduced or abolished Foxp3 production in Treg cells results in acquisition of effector T-cell properties to produce inflammatory cytokines [158–160]. Foxp3 is probably a major but not the master regulator of Treg cell [161] and indeed, it is not necessary for Treg cell development or functioning under certain conditions, for example, the lineage commitment of Treg cells in murine thymus does not require the expression of functional Foxp3 protein [162]. Activated purified naive CD4^+^ T cells transduced with a retroviral vector encoding Foxp3 and a Thy1.1 reporter produce a >95% Foxp3^+^ cell population but reproduce only a fraction of the Treg cell signature transcript [163]. Instead, other transcriptional regulators, for example, the combination of IL-2–STAT5 signaling and TGF-*β* or CD103 responding to Foxp3 play complementary and synergistic roles in controlling Treg cell signature gene expression [161]. Cytokines such as IL-2, TGF-*β* induce Foxp3 expression and also activate STAT5. The latter directly binds the promoter and the first intron of Foxp3 gene to promote Treg cell development. The loss of STAT5 activation abolished Treg cell differentiation [164–168]. However, Foxp3 can be induced in the absence of STAT5 in developing thymocytes, and the maintenance of Foxp3 expression in Treg cells is STAT5 independent [158]. Perhaps cytokine-induced STAT5 activation is not required in the development of CD4^+^CD25^+^CD122^+^GITR^hi^Foxp3^−^ Treg cell progenitor. Nevertheless, activated STAT5 plays a critical role in converting Treg cell progenitors into mature Treg cells [40, 137, 169]. Treg cell suppresses Th1 cell function through inhibition of IFN-*γ* transcription during Th1 priming without disrupting T-bet expression and Th1 programming. This suppression is either IL-10 dependent or independent depending on the target T-cell stage of activation and its tissue location [170].

Lineage commitment of Tfh cells is controlled by transcriptional factor Bcl-6, identified by the transcriptional profiles obtained from microarray analysis in Tfh cells that was Bcl-6 upregulated [171]. Bcl-6-deficient T cells were unable to differentiate into Tfh cells and could not sustain germinal center responses [172, 173]. Enhanced expression of Bcl-6 in CD4^+^ T cells promoted expression of Tfh cell signature molecules CXCR5, CXCR4, PD-1, and downregulated IFN-*γ* and IL-17 production [172] inhibited other Th lineage cell differentiation [173]. A transcriptional repressor, B lymphocyte-induced maturation protein 1 (Blimp-1) inhibits Tfh cell generation and function, indicating reciprocal regulation of Bcl-6 and Blimp-1 during Tfh cell differentiation [174]. STAT3 is necessary for Tfh cell development [104, 175]. Deletion of STAT3 in CD4^+^ thymocytes resulted in a greatly reduced number of differentiated Tfh cells after immunization. STAT3 deficiency in T cells also led to defective germinal-center B cell generation and antibodies production [104, 175]. Without STAT3, for example, blockage by a STAT3 inhibitor, even after being activated by IL-6, Tfh cells did not signal B cells [175, 176].

When exposed to foreign antigens, peripheral naive CD8^+^ T cells differentiate into two reciprocal subsets: short-lived effector T cells, that is, CTLs and long-lived memory T cells [177–179]. Memory T cells can be subdivided into central (Tcm) or effector memory T cells (Tem). Tcm cells express high levels of CCR7 and CD62L and lack immediate effector function but efficiently stimulate DCs in secondary lymphoid organs inducing a new wave of effector cells when secondary challenge occurs. Tem cells express low levels of CCR7 and CD62L, migrate to the infection site and produce cytokines and cytolytic molecules [177, 180]. Tem cells possess most features of CTL. However, Tem cells persist after the elimination of the invading pathogen [177]. A new memory T-cell subset with stem-cell-like properties has recently been identified and termed memory stem T cell (Tscm). This cell is present in humans [181] and mice [182]. Phenotypically within the naive T-cell compartment, for example, CD45RO^−^, CCR7^+^, CD45RA^+^, CD62L^+^, CD27^+^, CD28**^+^**and IL-7R*α* ^+^, human Tscm cells highly express CD95, CXCR3, Bcl-2, the *β* chain of the IL-2 and IL-15 receptor (IL-2R*β*) and lymphocyte function-associated antigen 1 (LFA-1). These cells possess the characteristics of memory T cells such as the ability to rapidly acquire effector functions upon antigen rechallenge. They also can secrete inflammatory cytokines in response to *α*-CD3/CD2/CD28 stimulation. Such Tscm cells represent the least differentiated T memory cell subset [181]. Wnt/*β*-catenin signaling may play a role in the induction of this subset [183] but there is conflicting evidence [184].

It has been suggested that the CD8^+^ effector and memory T cell develops from a single precursor cell when instructed by distinct TCR signals, cytokines [185–189] and not by the APC or when priming of T cell takes place [188]. Naive CD8^+^ T cells when primed by signals from TCR and co-stimulatory molecules differentiate into precursor cells or early effector cells expressing transcription factor T-bet and cytotoxic cytokines, for example, IFN-*γ*, tumor necrosis factor (TNF) *α* to acquire partial cytolytic abilities [177]. Whether the precursor cell further differentiates into late effector cell or memory cell is determined by a variety of factors such as the amount of IL-2R and IL-12 [190, 191], varying amounts of intracellular components, for example, T-bet, CD8, CD69, CD43, CD25, CD44, different expression of IFN-*γ*, Granzyme B, IL-7R*α*, and distinct granularity due to asymmetric division [187, 192]. Point mutations in the TCR *β* transmembrane domain block the development and function of CD8^+^ memory T cells. Yet primary effector CD8^+^ T-cell response is not affected by this mutation. Mutant T cells are unable to induce polarized TCR and intact NF-*κ*B signals in the immunological synapse (the interface between an APC and a lymphocyte). Therefore, distinct TCR signals trigger different programs for CD8^+^ T-cell differentiation toward either effector or memory pathways [186].

Transcriptional factors, T-bet, eomesodermin (Eomes), Bcl-6 and Blimp-1 are involved in CD8^+^ T-cell differentiation. T-bet is the master regulator of CD8^+^ T cells [178]. Its expression is responsible for IFN-*γ* production and it participates in the activation of cytolytic genes, for example, Granyeme B, Perforin expression of CD8^+^ T cell [193]. The presence of T-bet with a low level of IL-2 signaling is sufficient to induce CD8^+^ T cells to develop effector functions but other factors may also participate in terminal differentiation [194, 195]. Eomes, another member of the T-box family of transcriptional factors, is a key transcriptional factor for CD8^+^ T-cell differentiation [196]. T-bet and Eomes cooperate redundantly to induce effector CD8^+^ T-cell differentiation and can also act reciprocally to induce memory CD8^+^ T-cell development [197]. T-bet promotes the differentiation of short-lived effector CD8^+^ T cells at the expense of central memory cells and Eomes expression favors memory CD8^+^ T-cells differentiation [198, 199]. The differing quantities of T-bet in diverse T-cell lineages may be attributed to the asymmetric degradation [192]. Proteasomes are unequally distributed during asymmetric cell division and this is responsible for the imbalanced degradation of T-bet in the daughter cells resulting in differing allocation of T-bet to various cell lines [192].

Bcl-6 and Blimp-1 are transcriptional repressors. Blimp-1 expression is required for the terminal differentiation of effector CD8^+^ T cells, that is, the short-lived CD8^+^ CTLs [200–202]. Bcl-6 probably works as a reciprocal regulator of Blimp-1 in the process of CD8^+^ T-cell differentiation [203]. In general, lymphocytes with higher expression of Bcl-6 exhibit greater proliferative capacity, less secretory capacity and promote memory T-cell development. Lymphocytes with higher expression of Blimp-1 exhibit lower proliferative capacity and greater secretory capacity and they are more conducive to CTL development [203]. Blimp-1 is also highly expressed in exhausted CD8^+^ T cells [204]. T-bet can induce Blimp-1 transcription via enhanced IL-2R signaling [194].

STAT5 plays a critical role in the maintenance of phenotype of effector CD8^+^ T cells. It is also required in the induction of the anti-apoptotic molecule Bcl-2 expression by IL-7 and IL-15 and the maintenance of Bcl-2 expression in effector CD8^+^ T cells [205]. Constitutive STAT5 activation can promote effector and memory CD8^+^ T-cell survival and Bcl-2 expression [206].

## 3. TLRs Signaling

### 3.1. The TLR Family

Toll was initially identified as an essential protein that plays a central role in the establishment of dorsoventral polarity in the embryo of Drosophila [207, 208]. Later, it was recognized as a key modulator for the immune response against fungi in adult Drosophila [209]. Toll-receptor homologues have also been found to be capable of activating adaptive immune response through NF-*κ*B signal [12, 210]. As these receptors are evolutionally and functionally homologous with Drosophila Toll, collectively they are referred to as Toll-like receptors [210, 211].

Thirteen TLRs have been currently identified, TLR1 to TLR13, of which TLR1 to TLR9 are conserved both in human and mice. TLR10 is not functional in mice while TLR11, TLR12 and TLR13 are absent from human genome [212]. TLRs are type-1 transmembrane glycoproteins with a trimodular structure consisting of an N-terminal extracellular ectodomain characterized by inclusion of 16–28 leucine-rich repeats (LRRs), a transmembrane portion containing a single *α*-helix and an intracellular cytoplasmic portion with Toll/IL-1 receptor (TIR) domain [213, 214]. Each LRR region is composed of 24 amino acids with the conserved motif XLXXLXXLXLXXNXLXXLPXXXFX in sequence, an *α*-helix and a *β*-sheet connected by a loop in conformation [214, 215]. The LRRs of the ectodomain combine to display horseshoe-like shape. However, the LRR regions of TLR1, TLR2 and TLR4 do not have the typical conformation in that the conserved asparagine ladder in the central region of LRRs is absent. Consequently, this allows them to adjust their conformation to bind a variety of ligands and coreceptors for signaling [215]. The TIR domain is composed of a five-stranded *β*-sheet encircled by 5 *α*-helices. The B-B loop that connects *β*-strand B with *α*-helix B in the TIR domain is considered the essential structure for TIR dimerization and subsequent recruitment of TIR domain-containing adaptors [215].

TLRs can be classified as cell-surface TLRs or intracellular TLRs. The former group consists of TLR1, TLR2, TLR4, TLR5, TLR6, TLR10, TLR11 and TLR12, and it is largely expressed on the cell surface and recognizes molecules mainly from microbial membrane, for example, lipid, lipoprotein, or lipopeptide and protein. The latter group is composed of TLR3, TLR7, TLR8, TLR9, and perhaps TLR13 in mice localized in intracellular compartments like endoplasmic reticulum (ER), endosomes, lysosomes, and endolysosomes to detect microbial nucleic acids [212, 216]. The distinct ligand-sensing functions of the individual TLRs may explain their different localization. TLRs on cell surfaces mainly recognize molecules on the surface of the pathogenic microorganisms while those localized intracellularly sense nucleic acids which are released by intracellular degradation of the invading pathogen [217]. An advantage of the intracellular localization of nucleic-acid-sensing TLRs may be the avoidance of TLRs activation by the host homogeneous nucleic acid. Such nucleic acids released from the dying cells can be readily degraded by serum or cytoplasmic nucleotidases before their arrival to the endosome. As nucleic acid-sensing TLRs reside intracellularly, this prevents the occurrence of autoimmunity. However, viral nucleic acid is protected by the viral capsid proteins and is capable of staying in the endolysosome, being recognized by nucleic-acid-sensing TLRs to trigger antiviral immunity [217, 218].

### 3.2. TLR Signaling Pathway

**Figure 4:**
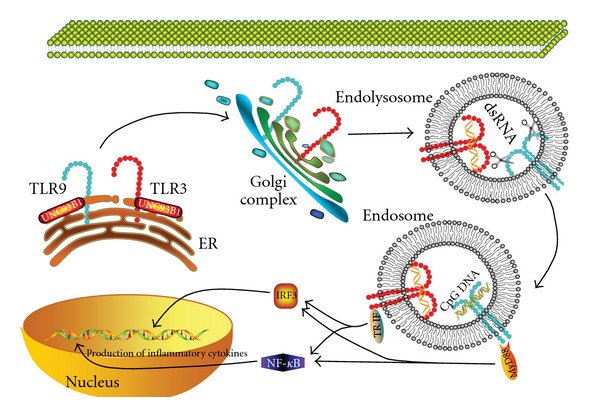
Intracellular TLRs traffic. Intracellular TLRs are present in the ER in resting cells and migrate to endosomes upon stimulation. Chaperone proteins, for example, UNC93B1 are required for their residence in ER and for their intracellular trafficking. When the ligands are taken into the cell, TLRs exit the ER through Golgi complex by conventional secretory pathways and reach the endolysosome where they interact with the ligands. TLR9 is cleaved by lysosomal cysteine proteases within their ectodomains in the endolysosome. TLR3 does not appear to be required for proteolysis during intracellular trafficking.

Intracellular TLRs are present in the ER in resting cells and move to endosomes upon stimulation of the cells (Figure 4). Their residence in ER is maintained by retention signals, for example, the cytoplasmic and ectodomains of TLR9 [219], a 23-amino acid sequence [Glu(727) to Asp(749)] present in the linker region between the transmembrane domain and TIR domain of TLR3 and the transmembrane region of TLR7 [220]. These TLRs can only be activated after being transported to endolysosome [217]. The trafficking of intracellular TLR9 from ER to endolysosomes is through traditional secretory pathways, and Golgi export is required for optimal TLR9 signaling [218, 221, 222]. Trafficking of TLR9 and TLR7 involves a cleavage by lysosomal cysteine proteases within their ectodomains. Without proteolytic modification, their association with myeloid differentiation protein 88 (MyD88) and subsequent signaling is disabled although the capacity of ligand-binding is preserved [216, 218, 221]. Proteolysis is not required for TLR3 signaling during its intracellular trafficking.

Chaperone proteins are required for maintaining the retention of these TLRs in ER in resting cells and their intracellular trafficking. UNC93B1, a highly conserved multiple membrane-spanning protein in ER, is involved in trafficking of nucleotide-sensing TLRs (Figure 4) [223]. A point mutation of UNC93B1 abolishes signaling of TLR3, 7, 9 and 13 as binding to their transmembrane domains is prevented [224]. Association with UNC93B1 promotes TLR9 signaling and represses TLR7-mediated response and mutation of the N-terminal D34A amino acid that suppresses TLR7 signaling enhances TLR7 trafficking and downregulates TLR9 trafficking in DCs. This suggests UNC93B1 favors DNA sensing but not RNA sensing. TLR3 signaling is promoted by overexpression of UNC93B1 and not affected by the N-terminal mutation [225]. However, a recessive N-ethyl-N-nitrosourea-induced mutation (triple D or 3d mutation) that is a missense allele of UNC93B1 disrupts exogenous antigen presentation and signaling via TLR3, TLR7 and TLR9 [226]. Therefore, UNC93B1 is essential for intracellular TLRs signaling and determines the trafficking efficiency of each individual TLR from ER to endolysosome to recognize the ligand and trigger subsequent response [216].

Upon binding ligands, TLRs dimerize to form homodimer or heterodimer (e.g., TLR2/TLR1, TLR2/TLR6 and perhaps TLR2/TLR10) and recruit adaptor molecules through the interaction of their intracellular TIR domain and the TIR domain of adaptor molecules [227]. Four adaptor molecules have been characterized. MyD88 [228] and TIR domain-containing adaptor inducing interferon-*β* (TRIF)/TIR domain containing adaptor molecule-1 (TICAM-1) [229, 230] are the two major adaptors for TLRs signaling. The remaining two adaptors, that is, TIR domain-containing adapter protein (TIRAP)/MyD88-adapter-like (Mal) [231, 232] and TRIF-related adaptor molecule (TRAM) [233], bridge the TIR domains between some TLRs and MyD88 or TRIF, respectively. MyD88 is a universal adaptor for all TLRs except for TLR3 and activates NF-*κ*B signal pathway to induce inflammatory cytokines. TLR3 and TLR4 use TRIF as their adaptor to activate interferon regulatory factor 3 (IRF3) and NF-*κ*B to promote the productions of type-I IFN and inflammatory cytokines. TIRAP/Mal is required for TLR4 and TLR2 signal transduction by bridging the TIR domain of TLR4 or TLR2 and MyD88 [215, 234]. Similarly, TRAM also acts as a bridging adaptor for TLR4 and TRIF [215].

**Figure 5:**
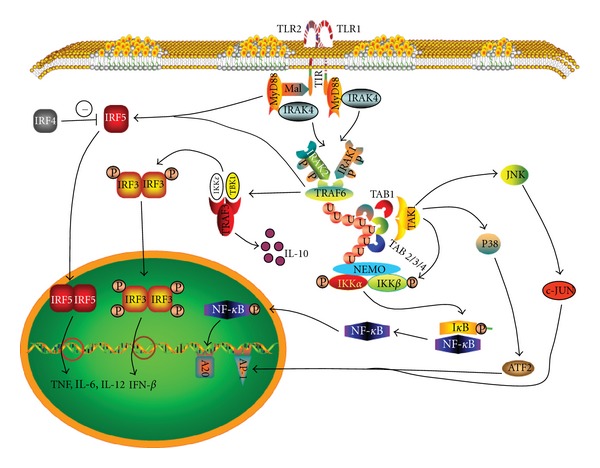
MyD88 signal pathway. MyD88 is the universal adaptor of all the identified TLRs except TLR3. In this figure, TLR1/TLR2 is used to illustrate the MyD88 signal pathway. TLR1/TLR2 uses triacryl lipopeptide as the ligand to recruit MyD88 via its cytoplasmic TIR domain. MyD88 interacts with DD to associate with IRAK4. IRAK4 then phosphorates IRAK1 and IRAK2 activates TRAF6. TRAF6 induces the synthesis of polyubiquitin chains that links TRAF6, NEMO, IRAK1 and TAB2, 3, 4. The ubiquitination of TAB2/3/4 in association with TAB1 activates TAK1. This induces phosphorylation of IKK complex resulting in the dissociation of I*κ*B and NF-*κ*B. NF-*κ*B then translocates into nucleus to induce the gene transcription of proinflammatory cytokines. TAK1 also activates JNK and p38 which induce AP1 activation. MyD88 and TRAF6 both activate IRF5 and induce proinflammatory cytokines. This activation is inhibited by IRF4. TRAF6 also interacts with TRAF3 and then recruits TBK1 to activate IRF3 and IFN-*β* production. TRAF3 alternatively induces the anti-inflammatory cytokine IL-10.

MyD88 is the essential adaptor for most TLRs. Upon ligand recognition, TLR recruits MyD88 to its cytoplasmic TIR domain by association with the TIR domain of the adaptor molecule (Figure 5). MyD88 possesses an N-terminal death domain (DD) that associates with DD of IL-1R-associated kinase-4 (IRAK4) [235]. IRAK1 and IRAK2 are phosphorylated by IRAK4 and then activate TNF receptor associated factor-6 (TRAF6) [236, 237]. TRAF6 acts as an E3 ubiquitin protein ligase to ubiquitinate itself and NF-*κ*B essential modulator (NEMO) by the formation of polyubiquitin chains. Both TRAF6 and NEMO are connected with IRAK1 by the chains. These chains also connect NEMO with the transforming growth factor *β*-activated kinase-1- (TAK1-) binding proteins (TABs) including TAB2, 3 and 4 which promote phosphorylation of TAK1-TAB1 resulting in TAK1 activation [238–241]. The activated TAK1 induces phosphorylation of I*κ*B kinase-related kinase (IKK) *β*. This causes I*κ*B phosphorylation and its dissociation with NF-*κ*B. Consequently, the nuclear translocation of NF-*κ*B is induced and this culminates in the transcription of proinflammatory cytokines, for example, TNF and IL-6. The TAK1/TABs complex also phosphorylates and activates c-Jun N-terminal kinase (JNK) and p38 resulting in activation of activator protein 1 (AP1) [216, 227]. IRF5 can be activated by both MyD88 and TRAF6, and it promotes the transcription of proinflammatory cytokines [242]. This can be inhibited by the competition by IRF4 [243]. TRAF6 also induces TRAF3 triggering noncanonical TRAF3 self-ubiquitination [244] and this complex associates with TRAF family-member-associated NF-*κ*B activator-binding kinase 1 (TBK1). It then acts with IRF3 to induce IFN-*β* production. Ubiquitinated TRAF3 also induces the anti-inflammatory cytokine IL-10 [245, 246]. In plasmacytoid DCs (pDCs), MyD88 signaling elicited by TLR7 and TLR9 is different from that in myeloid DCs (mDCs). Through phosphatidylinositol 3-kinase (PI3K), MyD88 signaling in pDCs ultimately activates IRF7 to induce production of enormous quantities of IFN-*α* [247–249]. In humans, TLR3 is predominantly expressed in mDCs whereas TLR7 and TLR9 are exclusively expressed in pDCs [250–255]. TLR expressions in murine DCs are not restricted as seen in human DCs. In mice, mDCs (alternatively named conventional DCs, cDCs) express all TLRs except TLR7 which is not expressed by CD8*α* ^+^ mDCs [250, 256]. Indeed, murine pDCs highly express TLR7 and TLR9 along with mRNAs of all the remaining identified TLRs. TLR3 is preferentially expressed in CD8*α* ^+^ mDCs and possibly not expressed in pDCs [250, 256]. Therefore, effective antitumor immunity elicited by CpG DNA in mouse is not seen in humans [257].

**Figure 6:**
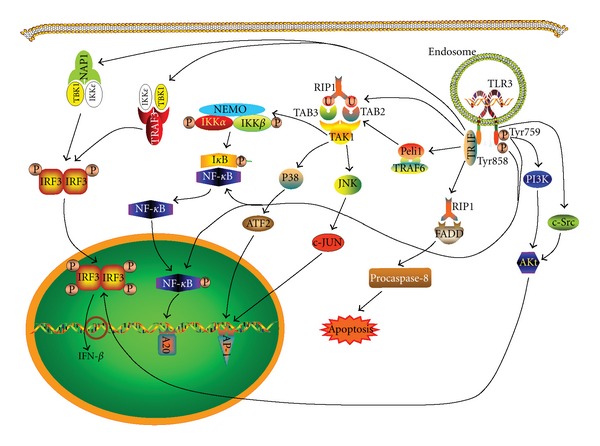
TRIF signal pathway. In TLR1-TLR13, TRIF is the sole adaptor of TLR3 and also an adjunct adaptor of TLR4. Here, the TLR3-TRIF signal is illustrated as an example of TRIF pathway. dsRNA that is internalized in endosome binds to TLR3, which possesses two dsRNA binding sites near the N-terminus and C-terminus, respectively. When combined with dsRNA, a sole dsRNA molecule associates two TLR3 molecules through four dsRNA binding sites in an “m” shape. TLR3 TIR domain combines with the TIR domain of TRIF. The interaction of TRIF with RIP1 or TRAF6 and Peli1 results in polyubiquitination of RIP1, the latter binds ubiquitin receptors TAB2 and TAB3 which activates TAK1. Activated TAK1 induces phosphorylation of IKK complex composed of IKK*α* and IKK*β* and NEMO. This results in the degradation of I*κ*B which ultimately causes the nuclear translocation of NF-*κ*B to activate the specific gene promoter A20. TAK1 also interacts with JNK and p38 to activate c-JUN and ATF2. This results in the activation of the AP-1 transcription factors family. TRIF also activates TBK1 and IKK*ε* through NAP1 inducing phosphorylation and nuclear translocation of IRF3 culminating in IFN-*β* production. TRAF3 binds with the TBK1/IKK*ε* complex inducing IRF3 activation. Combination of TRIF results in phosphorylation of Tyr759 and Tyr858 in the TLR3 TIR domain which subsequently induces the phosphorylation and degradation of I*κ*B leading to NF-*κ*B release. Phosphorylated Tyr759 recruits PI3K and phosphorylates kinase Akt and activates nucleic IRF3. Tyrosine kinase c-Src also plays a role in Akt activation. The unique signaling of TRIF is that it interacts with FADD through RIP1 and activates procaspase-8 to initiate cell apoptosis.

TRIF is the sole adaptor of TLR3 and the adjunctive adaptor of TLR4. After sensing dsRNA, the TIR domain of TLR3 associates TRIF TIR, then TRIF interacts with receptor-interacting protein 1 (RIP1) through the RIP homotypic interaction motif (RHIM) present in both proteins (Figure 6). TRAF6 is also recruited to the N-terminal domain of TRIF followed by polyubiquitination of RIP1. Peli1, a member of Pellino family of RING-like domain-containing E3 ubiquitin ligases, also participates in RIP1 polyubiquitination along with TRAF6 [258]. The polyubiquitinated RIP1 recruits the ubiquitin receptor proteins TAB2 and TAB3, which in turn activate TAK1 [259]. TAK1 then phosphorylates IKK*α* and IKK*β* leading to degradation of I*κ*B which results in the translocation of NF-*κ*B to cell nucleus to stimulate proinflammatory cytokine production [260]. Similar to MyD88 signaling, TAK1 activates AP1 through JNK and p38. TRIF also associates its adaptor protein NF-*κ*B activating kinase- (NAK-) associated protein 1 (NAP1) to activate TBK1 and IKK*ε* resulting in the phosphorylation and nuclear translocation of IRF3, inducing the expression of IFN-*β* [261]. TRAF3 combines with the TBK1/IKK*ε* complex and is also involved in the TRIF-mediated IRF3 activation [245]. It is a unique signal pathway of TRIF that interacts with Fas-associated cell death domain (FADD) protein through RIP1 which in turn activates procaspase-8 to initiate cell apoptosis [262, 263]. Recently, a TIR-less splice variant of TRIF (designated as TRIS) was found capable of activating IRF3 through the interaction with TBK1 and/or activating NF-*κ*B via RIP1 [264]. TLR3 itself is also involved in signaling, for example, the phosphorylation of Tyr759 and Tyr858 in the TLR3 TIR domain. Phosphorylated Tyr759 recruits PI3K to activate kinase Akt which in turn activates IRF3 in nucleus [265]. Additionally, the phosphorylation of Tyr759 and Tyr858 induces degradation of I*κ*B to release and partially activate NF-*κ*B by phosphorylation [266]. Tyrosine kinase c-Src also involves Akt activation [267].

## 4. Effects of TLR Activation on T Lymphocyte Subsets Differentiation

### 4.1. TLR Signals Affect Thymocytes Differentiation

Various viral infections through TLR interaction can induce type I IFN production. TLR3 recognizes ssRNA virus (West Nile virus), dsRNA virus (reovirus), respiratory syncytial virus, mouse cytomegalovirus (MCMV); TLR7 recognizes ssRNA viruses (vesicular stomatitis virus, influenza virus); TLR8 recognizes ssRNA from RNA virus; TLR9 recognizes dsDNA viruses (Herpes simplex virus, MCMV), CpG motifs from bacteria and viruses [268, 269]. Treatment of newborn mice with an active IFN-*α*2/*α*1 hybrid molecule reduced thymus cellularity by 85%. Phenotypic analysis revealed that the quantity of CD44^+^CD25^−^ DN1 cells increased while that of CD44^−^CD25^−^ DN4 cells decreased suggesting that the IFN-*α*2/*α*1 inhibition of T-cell development begins at an early progenitor stage [270]. There are deleterious effects of IFN-*α* on T-cell development mediated by upregulation of cyclin-dependent kinase inhibitor p27^Kip1^ [271]. The TLR3 ligand polyinosinic-polycytidylic acid (poly(I:C)) and TLR7 ligand loxoribine are capable of inducing type I IFN expression resulting in a decrease in CD44^−^CD25^+^ DN3 population [272]. Poly(I:C) can block the DN1-DN2 transition, diminish the DN3-DN4 cell proliferation, promote apoptosis of DP thymocytes, which culminate in a reduced thymic output [273]. As poly(I:C) can activate the cytoplasmic helicases RIG-I and melanoma differentiation-associated gene 5 (MDA-5) pathways [260], the inhibitory effects of poly(I:C) on T-cell development may be not solely mediated by TLR3. Activation of MDA-5 causes a reduction in thymus size while TLR9 ligand CpG DNA and TLR4 ligand lipopolysaccharide (LPS) did not reduce thymus size [274]. Upon stimulation by LPS, the gene expression of downstream signals of TLR3 and TLR4, that is, TRIF signal, is the most differentially affected pathway in murine thymocytes, suggesting a direct influence of altered TLR signaling on thymus involution [275].

### 4.2. Effects on T-Cell Differentiation through TLR Activation in APC

TLRs activation has been shown to bridge the innate and adaptive immunity [212, 276–278]. Beside its expression in professional APCs such as DCs and macrophages [276], TLRs can be expressed in T cells [254, 279, 280] and serve as co-stimulatory signals in T-cell activation [268, 277, 278, 281]. Traditionally, activation of TLRs in APCs would lead to the production of IFN-*α*, proinflammatory cytokines such as TNF-*α*, IL-1 and IL-6, and the cytokines IL-12 and IL-18 that instruct Th1 to differentiate, whereas an increased Th2 response was observed in MyD88 deficient mice with impaired TLRs signaling [282–284]. The IL-12 and IL-23 secretions of DCs induced by TLRs activation are enhanced by chemokine CCL17 in an autocrine manner. The productions of these cytokines are significantly reduced in CCL17-deficient DCs [285]. It has been demonstrated that the dose of antigen plays an important role in directing Th1/Th2 differentiation driving by DCs. A lower concentration of ovalbumin (OVA) peptide (1 and 10 ng/mL) induced Th2 commitment while higher concentrations (1 *μ*g/mL and 100 ng/mL) failed to elicit Th2 development. Stimulation of CD4^+^ T cells with DCs along with TLR2 or TLR9 agonists in the presence of the 10 ng/mL of OVA peptide, the optimal antigen concentration for Th2 development resulted in suppression of IL-4 production and Th2 development. This suggests that TLR-activated DCs can block Th2 lineage commitment independent of antigen dosage [286]. A lower dose of LPS (0.1 *μ*g), through TLR4 signaling, induced a Th2 response to inhaled antigens in a murine allergic sensitization model. In contrast, high doses of LPS (100 *μ*g) with antigen resulted in a Th1 response [287]. However, repeated administration of TLR2 ligand Pam_3_CSK_4_ or TLR4 ligand LPS leads to tolerance of TLR2 [288] or TLR4 [289] with reduced cytokine release and expression of IRAK-1 and IRAK-4 proteins [288]. Additionally, activation of TLR4 resulted in a MyD88-dependent Th17 response in memory CD4^+^ T cells in the absence of TRIF molecule [284]. Activation of DCs via TLR2-MyD88 also induced Th1 and Th17 cell differentiation [290]. Still, signaling of TLR2 can inhibit DCs to produce IL-12p70 by dampening the type 1 IFN amplification loop. This signaling also drives the immune response induced by synergistic combination of TLR4 and TLR7/8 agonists (both are potent inducers of Th1 responses) toward Th2 and Th17 responses in the naive and memory T-cell subpopulations [291]. Murine DCs activated by LPS or CpG oligodeoxynucleotide (ODN) overcame Treg-mediated suppression by inducing IL-6 signals [292]. IL-6 also mediates the downregulation of Foxp3 expression in T cells induced by TLR7-activated DCs [293]. However, activation of TLR7 by resiquimod in OVA-induced experimental model of murine allergic asthma resulted in expansion of Treg cell through a TGF-*β*-dependent pathway [294]. Thus, it seems that T-cell subsets activated by TLR signals from APCs vary depending on the type and the status of APC involved, the cytokine milieu, as well as the amount of the antigen present [295–297].

On the other hand, a recent report indicated that signals from Th cells can govern the formation and function of specialized DC subsets, for example, Th1 and Th17 cells cause monocytes differentiation into Th1- or Th17-promoting DC subsets in psoriasis lesion, and Th2 cells induce the production of Th2-promoting DC subset in acute atopic dermatitis [298]. The phenotype of these polarized DC subsets cannot be altered even after subsequent stimulation of TLR ligands. With stimulation by ligands of TLR1-TLR9, the quantities of cytokine secreted by the specialized DC subset were changed but the overall cytokine secretion profile remained the same [298]. The TLR signaling in DCs is negatively regulated by adapters containing immunoreceptor tyrosine-based activation motif (ITAM) sequences to suppress activation of DCs [299], for example, DNAX-activating protein of molecular mass 12 kilodaltons (DAP12) in mDCs [300] and Fc receptors for IgG in pDCs [301]. The triggering receptor expressed on myeloid cell-2 (TREM-2) associates DAP12 to suppress TLR signaling in bone-marrow-derived DCs [302]. The ligand of TREM-2 is also detected on the surface of these DCs. Thus, it seems that the preexisting polarized immunity dictates that the subsequent immune response and this polarization will not be altered even if stimulated by PRR.

### 4.3. Direct Activation of TLR in CD4^+^ T Effector Cells Induces Costimulation

The expression and the activity of TLRs in T cells are related to the functional status, for example, effector or memory cells and central memory or effector memory cells as well as the activation status of T cells by TCR signals (Table 1) [268, 277, 303]. Murine naive T cells can express TLR1-TLR9 although there is a considerable variation in expression levels [303]. TLR1, TLR4 and TLR6 were among those maximally expressed in CD4^+^ and CD8^+^ T cells [277]. Although naive human CD4^+^ T cells express significant levels of intracellular TLR2 and TLR4 protein, cell surface expression of TLR2 and TLR4 was found only in activated CD4^+^ T cells [281]. Cell surface expression of TLR2 in CD4^+^CD45RO^+^ (memory) T cells is significantly higher than that of CD4^+^CD45RA^+^ (naive) T cells. However, TLR2 expression by naive T cells can be significantly increased by anti-CD3 activating TCR. This is enhanced by TLR2 ligand. An activation marker, HLA-DR antigen, was found coexpressed with TLR2 in parallel suggesting that TLR2 expression is associated with T-cell activation [281]. Similar results were also obtained in CD8^+^ T cells with transcript copies of TLR2 mRNA in CTLs 7–10 times higher than that in naive CD8^+^ T cells [304]. However, TLR expression in T cells is controversial. When poly(I:C) and CpG DNA were added to murine CD4^+^ T-cell cultures that were TCR activated by anti-CD3 antibody, TLR3 and TLR9 expression was upregulated with enhanced survival. By contrast, levels of TLR2, TLR4 were undetectable when peptidoglycan and LPS were used [305]. Activated murine CD4^+^CD25^−^ effector T cells can functionally express TLR2 [306]. The discrepancy may be attributed in part to the different protocols used for T-cell purification and the different ligands used for TLR activation. A study compared the differences in purity, activation requirements, specifically, the response to TLR ligands of human CD4^+^ T cells isolated by immunomagnetic cell sorting (IMACS-CD4^+^) or by IMACS followed by fluorescence-activated cell sorting (FACS, IMACS/FACS-CD4^+^) [307]. It showed that the IMACS/FACS-CD4^+^ T cells were highly purified (99.7%) and when stimulated by TLR4 ligand LPS, in the absence of TCR activation by anti-CD3 and costimulation from anti-CD28, did not elicit a response. On the other hand, a less pure sample of IMACS-CD4^+^ T cells (92.5%) showed IL-2 and IFN-*γ* secretion responding to anti-CD3 without anti-CD28. Stimulation with anti-CD3, anti-CD28 and LPS significantly increased proliferation and cytokine production of IMACS-CD4^+^ but not IMACS/FACS-CD4^+^ T cells. The expression of TLR4 was also significantly higher in IMACS-CD4^+^ cells than in IMACS/FACS-CD4^+^ cells. This difference is likely to be the result of contaminating accessory cells in IMACS-CD4^+^ population [307]. Another report using LPS derived from *Salmonella enteritidis*, *Salmonella minnesota* and *Salmonella typhimurium* demonstrated that only LPS from *Salmonella typhimurium* can induce proliferation and IFN-*γ* secretion in murine CD4^+^ T cells [306].

**Figure 7:**
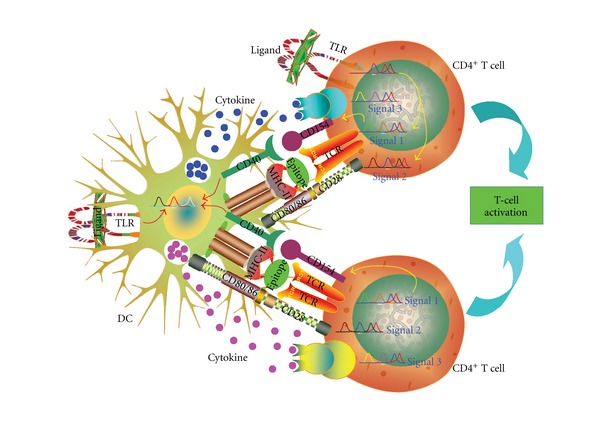
Costimulation of T cells. Antigen uptake by DCs is followed by epitope presentation by MHC complex molecules to TCR expressed on T-cells surface (signal 1). Upon TCR-activation signal, T cells produce CD154 to bind CD40 on the cell surfaces of DCs to further activate DCs. After interacting with TLRs, DCs express CD80 and CD86 which combine with CD28 in T cells for costimulation of T cells (signal 2). Activated DCs also produce cytokines to instruct T cells for polarized differentiation (signal 3). TLRs expressed in T cells act as co-stimulatory molecules in T-cell activation by reducing the activation requirements for signals 1 and 2 and generating efficient memory T cell in response to a weak signal 1. Some TLR ligands even can induce signal 2 in the absence of CD28 via activation of TLR expressed on T cells.

TLRs expressed in T cells have been suggested to act as co-stimulatory molecules involved in T-cell activation [268, 277]. Application of Pam_3_CysSK_4_, the ligand of TLR1/TLR2 complex, in activated TCR transgenic mice CD8^+^ T cells resulted in increased cell proliferation and survival. This was associated with a sustained CD25 expression and an enhanced expression of Bcl-xL, an antiapoptotic molecule. TLR2 engagement also enhances production of IFN-*γ* and granzyme B, promotes cytotoxic activity of antigen-activated CD8^+^ T cells, reduces the activation requirements for co-stimulatory signals from APC and TCR signal strength, and generates efficient memory T cells in response to a weak TCR signal [308, 309]. TLR2 engagement on CD8^+^ memory T cells is also involved in the direct control of memory cell proliferation and IFN-*γ* production [310]. The co-stimulatory role of TLR2 ligation on CD8^+^ T cell is believed to be due to the intrinsic TLR2-MyD88 signaling and PI3K-Akt pathway activation in CD8^+^ T cells [308, 311]. PI3K signal activated by MyD88 adaptor is indispensable to the costimulation of CD4^+^ T cells by TLR9 ligand CpG ODN [312]. Costimulation by poly(I:C) of naive CD4^+^ T cells through TLR3 in the presence of anti-CD3 and anti-CD28 can induce synthesis of IL-17A and IL-21, this being dependent on activation of the NF-*κ*B pathway. IL-17A and IL-21 cause naive CD4^+^ T-cell differentiation toward an IL-21 phenotype. These cells do not have the transcription factors T-bet, GATA-3 and ROR-c that represent the induction of Th1, Th2 and Th17 subsets, respectively [313] and consequently such cells are absent. TLR ligands can act directly on highly purified T cells in the absence of CD28 engagement [303] but is unable to induce functional responses in naive T cells without concurrent TCR stimulation [308]. Therefore, TLR-induced signals in T cells are strictly co-stimulatory [303] (Figure 7).

### 4.4. Effects of Direct Activation of TLR on Treg Cells

TLR2 agonist Pam_3_Cys acts directly on purified Treg cells resulting in an augmented Treg cells proliferation. This is accompanied by a temporal loss of the suppressive Treg phenotype in the presence of TCR stimulation [314] and a transient suppression of Foxp3 expression [306]. The effects of a reversal of suppression on responder T cells by human CD4^+^CD25^+^Foxp3^+^ Treg cells influenced by the TLR2 ligand were Akt being phosphorylated and p27^Kip1^ (The cyclin-dependent kinase inhibitor which is highly expressed in Tregs and capable of arresting cell-cycle in the G1 phase, and can be reduced by IL-2) being downregulated. There was no alteration in Foxp3 expression [315]. On the other hand, engagement of TLR2 resulted in human CD8^+^CD25^+^Foxp3^+^ Treg cells expansion that directly suppressed CD4^+^ T-cells proliferation by cell-contact inhibition and triggered CD4^+^CD45RO^+^ memory T-cell apoptosis inhibiting allergen induced Th2 immune responses [316]. Treg cells are able to regain their suppressive property in the presence of IL-2 once the TLR2 ligand is removed [306, 314]. Although TLR2-stimulated Treg cells readily lost their ability to suppress proliferation of effector T cells, cytokine production by effector T cells was still repressed. This suggests that the activity of Treg cells was cytokines independent [317]. Treg and Th17 cells are considered divergent and mutually inhibitory. It has been reported that when naive CD4^+^ T cells were stimulated with TLR2 agonists Th17 differentiation *in vitro* and Th17 cytokine production occurred [318]. Thus, the reduced suppressive function of Treg cells induced by TLR2 stimulation may be a result of imbalanced phenotype and function between Treg and Th17 [315]. The suppression seen in both CD4^+^CD25^hi^Foxp3^low^CD45RA^+^ naive and CD4^+^CD25^hi^Foxp3^hi^CD45RA^−^ memory or effector Treg cells on CD4^+^CD25^−^Foxp3^−^CD45RA^+^ naive responder T cells can be reversed by activated TLR1/2. This is accompanied by increased production of IL-6 and IL-17, upregulation of ROR-c and downregulation of Foxp3 expression [319]. Pam_3_Cys-mediated reduction of Treg suppressive function can be abrogated by neutralization of IL-6 or IL-17 [319]. All together, in a bacterial infection, the TLR2 ligand augments the functional activities and the clonal expansion of effector T cells as well as temporarily attenuating the suppressive function of Treg cells against the invading pathogen. The TLR2 signal also promotes the expansion of Treg cells that have reduced suppressive function. As the TLR9 ligand can reprogram Treg population toward Th17 differentiation [320, 321], it is conceivable that TLR2 may play a role in Treg cell reprogramming. The proinflammatory cytokines IL-6 and IL-1*β* are crucial reprogramming cytokines of Treg cells toward Th17 differentiation [322, 323]. When a pathogen is eliminated, the expanded clusters of Treg cells recover their suppressive activity preventing autoimmunity that may result from over activated effectors (Figure 1) [303, 306, 324]. However, it is not known whether the changes observed in reprogrammed Treg cells can be reversed.

Pam_3_CSK_4_, a TLR1/TLR2 ligand can induce tumor remission in severe combined immunodeficiency (SCID) mice by diminishing the suppressive function of Foxp3^+^ Treg cells and enhancing the cytotoxicity of tumor-specific CTLs. Adoptive transfer of CTLs and Treg cells pretreated with Pam_3_CSK_4_ from wild-type mice into tumor-bearing SCID mice can restore antitumor immunity in SCID mice by reciprocal downregulation of Treg cells and upregulation of CTL function [325]. However, treatment of CD4^+^CD25^+  ^Treg cells with intrinsic TLR2 agonist, heat shock protein (HSP) 60, before anti-CD3 activation significantly enhanced the suppressive ability of the Treg cells to inhibit CD4^+^CD25^−^ or CD8^+^ T-cell proliferation, IFN-*γ* and TNF-*α* secretion [326]. Nevertheless, the purity of CD4^+^CD25^+^  Treg cells used being >90% implies possible contamination of other cell types. Not all the CD4^+^CD25^+^Foxp3^+^ cells from peripheral blood activated by HSP60 are Treg cells. Activated CD4^+^ effector T cells can also transiently express Foxp3. It should be noted that only cells with CD4^+^CD25^+^Foxp3^+^CD30^+^ phenotype possess suppressive function. This induction of Treg cells by HSP60 is enhanced by signaling via TLR4 on APCs [327]. Thus, contaminated APCs within the Treg cell population may promote the suppressive function of Treg cells by TLR4 signaling triggered by HSP60 in APC rather than by TLR2 signaling in Treg possibly accounting for this discrepancy. Indeed, TLR2 expression in human CD4^+^CD25^+^CD127^−^ Treg cells isolated from peripheral blood mononuclear cells is not present [328].

Activation of TLR4 in CD4^+^CD25^+^ Treg cells by LPS, in the absence of APC, can directly induce Treg cells activation. This activation involves the upregulation of activation markers, for example, CD69, CD44, CD38, as well as B7-1 and promotes cellular survival and proliferation [329]. TLR4 expression can be detected in peripheral human CD4^+^CD25^+^ Treg cells. Co-culture of these Treg cells with LPS induced activation of Treg cells with decreased expression of Foxp3. These cells repressed neutrophils in an IL-10- and TGF-*β*-dependent manner [330]. However, the enhancement of Treg cell function by LPS was not reproduced by other investigators [306, 314, 331]. It is possible that potential contamination of commercial LPS preparations with TLR2 ligands [314] or the presence of impurities of the cells [332] may create discrepant results [306, 314, 331]. Application of TLR5 agonist flagellin augments the suppressive capacity of CD4^+^CD25^+^ Treg cells with enhanced expression of Foxp3. CD4^+^CD25^+^ Treg cells can suppress effector T cells in a ratio of 1 : 81 and this inhibition was increased to 1 : 243 with the addition of flagellin [331]. TLR8 is exclusively expressed in human Treg cells, and triggering of TLR8-MyD88-IRAK4 signaling pathway can reverse the suppressive function of Treg cells [333]. A co-stimulatory effect of CpG DNA on CD4^+^CD25^−^ effector T cells is to abrogate the suppression by Treg cells [334]. CpG DNA can also directly act on CD4^+^CD25^+^ Treg cells to inhibit its suppressive effects [334]. Thus, the direct effect of individual TLR ligand on Treg cell is completely different although almost all of the TLR signals share a common pathway (Table 1).

Treg cells' phenotypic plasticity is seen by their expression of proinflammatory cytokines such as IL-17, IFN-*γ*, or IL-2 under certain conditions and their reprogramming into Th-like cells [321, 322]. Mice systemically administering high doses of CpG ODN at 50–100 *μ*g/mouse show activation of naive Treg cells in the spleen to acquire potent suppressor activity. This was mediated by the immunoregulatory enzyme IDO in pDCs. When IDO was blocked, CpG treatment stimulated pDCs to express IL-6 which in turn reprogrammed Foxp3 lineage Tregs to express IL-17 to become Th17-like effector T cells [335, 336]. The converted Treg cells play a helper role essential for initial priming of CD8^+^ T cells to a new cross-presented antigen. This was CD40L dependent. This process, unlike the help from conventional non-Treg CD4^+^ cells, did not require preactivation or prior exposure to antigen [320]. CD4^+^Foxp3^+^ Treg cells can also be reprogrammed into Tfh lineage in mouse Peyer's patches under the interaction with B cells and loss of Foxp3 expression [337]. Although the reprogramming of Treg cell has been recognized to play a critical role in the initiation of certain innate immune responses by vaccination with a TLR agonist adjuvant, that is, CpG ODN [320, 321, 338, 339], the effects of the activation of other TLRs besides TLR9 on reprogramming of T cells especially Treg cells are not known.

### 4.5. Modulation of CD8^+^ T-Cell Response by TLR Activation

Viral antigen taken up by APCs are processed into epitopes, loaded onto MHC-I molecules and cross-presented to CD8^+^ T cells eliciting an anti-virus CD8^+^ T-cell response. However, not all the potential epitopes can be equally cross-presented to CD8^+^ T cells. The epitopes recognized by the most abundant cognate T-cell populations are referred to as being immunodominant, while those recognized by less abundant T-cell populations are named as subdominant determinants. Thus, the immunodominant and subdominant determinants constitute a hierarchy (*α*-, *β*-, etc.) in an antiviral immune response [340]. This can be altered by TLR signals. Combined activation of TLR2 and TLR3 by Pam_3_cysk_4_ and poly(I:C) at the infection site of lymphocytic choriomeningitis virus (LCMV) in mice reduced antigen uptake and cross-presentation of an immunodominant determinant of LCMV, NP396 and shifted it becoming a subdominant determinant. However, administration of TLR4 ligand LPS did not induce this shift [341]. Therefore, combined activation of multiple TLRs could possibly induce a complex response instead of being merely synergistic or antagonistic.

### 4.6. Effects of TLR Activation on Peripheral T-Cell Tolerance

The outcome of presentation by DCs depends on its activation status. DCs activated by PAMPs, for example, TLR ligands from invading pathogen will be capable of producing co-stimulatory molecules and proinflammatory cytokines immunogenic. On the other hand, self-antigen from apoptotic self-cells lack TLR ligands and cannot induce maturation of DCs and this eventually results in tolerance [342, 343]. However, a tumor-associate antigen NY-ESO-1 was able to induce T-cell dependent antibody response through activation of TLR4 on DCs [344]. In addition, mature DCs induced by distinct stimulation may function differently. A recent study suggested that LPS matured DCs produced IL-12 to promote CD8^+^ T-cell trafficking and inflammation, whereas poly(I:C) matured DCs facilitate CD8^+^ T-cell infiltration and autoimmunity in an IFN-*α*-dependent manner [345]. Mesenchymal stem cells can inhibit DCs activation induced by LPS, block DCs migration to draining lymph node and impair its capacities to prime CD4^+^ T cells and cross-presentation to CD8^+^ T cells [346]. The cross-talk between different DC subsets is also important. The cDCs are indispensable for cross-presentation of cancer antigens in eliciting potent anticancer immunity. The efficacy of CpG in anticancer immunotherapy is dependent on activation of TLR9 in pDCs. CpG-activated pDCs induce upregulation of co-stimulatory molecule CD80 in cDCs, thus providing an adjuvant effect in anticancer immunotherapy [347]. Some specific DC subsets may be primarily tolerogenic even if activated. For example, a prototypic DC subset, Langerhans cells is found precommitted tolerogenic and unable to translocate RelB, an NF-*κ*B family member, to the nucleus [348]. However, although Langerhans cells are tolerogenic to bacteria without cell surface expression of TLRs, they can effectively sense virus and poly(I:C) to induce naive CD8^+^ T-cells expansion and differentiation into effector cells that are dependent on high expression of CD70 rather than mediated by IL-12 [349]. Therefore, mature DCs are not a homogenous population and instead a cell family with increasing new subset member being discovered [350]. They may function divergently depending on its activation status [351] and other factors such as the quality of stimulation, the communication between different DC subsets and the nature of DC subset.

Human monocytes, when cultured with Wnt5a and subsequently stimulated by TLR ligands, can differentiate into DCs. Enhanced production of inhibitory ligands PD-L1 and PD-L2 rather than upregulation of CD83, HLA-DR, CD40, CD86, CD80 and CCR7 molecules would also occur [352]. Additionally, these cells secrete low levels of IL-12p70 and TNF-*α*, however, there is an increased production of regulatory cytokine IL-10 with a reduced capacity of Th1 response. This tolerogenic DC induction by enhanced Wnt signaling is *β*-catenin independent but is dependent on noncanonical Ca^2+^/calmodulin-dependent protein kinase II/NF-*κ*B signaling [352]. Lymph node cells that have precommitted tolerant of self-antigen proteolipid protein, when stimulated by both CpG ODN and this protein, divided and differentiated into Th1 cell lineage. This is IL-12 dependent and these cells are capable of inducing autoimmune encephalomyelitis when they are transferred into naive mice [343]. The break of this cross-tolerance depends on the specific CD4^+^ T-cell help and stimulation by sole TLR ligands without the help from CD4^+^ T cell is insufficient to overcome this tolerance [353]. By contrast, induction of TLR signaling in T cells may increase tolerance. T-cell intrinsic TRAF6 is essential in the maintenance of peripheral tolerance. Deletion of TRAF6 in T cells leads to hyperactivation of PI3K-Akt pathway and increased resistance of T effector cells to the suppression by CD4^+^CD25^+^ Treg cells. This finally results in multiorgan inflammatory disease [354]. As TRAF6 is an important adaptor in TLR signaling, it is conceivable that activation of TLRs expressed in T cells may involve in maintenance of T-cell susceptibility to Treg cells via TRAF6.

Administration of TLR3 ligand poly(I:C) results in a strong expression of PD-1 ligand (PD-L1) in all subsets of LNSCs [56]. This may prevent the tolerized T cells in lymph nodes regaining their effector function. However, this also implies that a virus infection in LNSCs such as FRCs would not be eliminated hence becoming a persistent infection [355]. Activation of TLR3 by poly(I:C) also induces upregulation of MHC-I and co-stimulatory molecules in LNSCs, for example, CD80 and CD86 in FRCs, CD80 in LECs [56]. The net result of promoting immune response by enhanced expression of MHC-I and co-stimulatory molecules and promoting tolerance by augmented expression of PD-L1 is a decreased ability of FRCs to stimulate T-cell division in the presence of poly(I:C). However, the phenotypic alterations of these FRCs in PD-L1, MHC-I and co-stimulatory molecules such as CD80 and CD86 are similar to the DCs being treated by poly(I:C) [56]. The decreased stimulatory ability of these FRCs is considered to be the consequence of deduced production of specific antigen by FRCs [56]. Alternatively, this varying stimulatory capacity between FRCs and DCs may be due to the altered TLR signaling cascades in FRCs being tolerogenic cells [356].

The discrimination of self or nonself antigen by DCs is also TLR dependent [342]. TLRs control the TCR ligand generation in phagosome autonomously. With the conjugation of TLR ligand, the phagocytosed antigen by DCs can be selectively loaded on MHC-II molecules and preferentially presented in the context of costimulation [342]. Activation of TLRs is helpful to break tolerance in immunocompromised individuals. Blockade of CTLA4 or PD-1 in combination of TLR9 agonist CpG ODN treatment overcomes immune tolerance in tumor bearing mice with improved long-term survival, increased tumor-specific effector T-cell population and decreased Treg cell levels [357].

### 4.7. Effects of TLR Activation on Mucosal Tolerance

TLRs are directly involved in mucosal tolerance development. PAMPs from nonpathogenic commensal microorganisms in mucosa are also termed microbe-associated molecular patterns (MAMPs) [358].

TLR1, TLR2, TLR3, TLR4 and TLR5 as well as TLR9 proteins have been found expressed both in human small intestines and colon [359]. However, their expression and action in enterocytes are different even within the same cell. Activation of TLR9 through apical and basolateral surface domains of intestinal epithelial cell (iEC) results in distinct transcriptional responses. Basolateral activation of TLR9 induces I*κ*B*α* degradation and activation of the canonical NF-*κ*B signal pathway. Apical TLR9 stimulation elicits a unique response with accumulation of ubiquitinated I*κ*B*α* in cytoplasm-suppressing NF-*κ*B activation. This results in intracellular tolerance to subsequent TLR9 basolateral challenge. It also blocks apical TLR2 and basolateral TLR3 or TLR5 stimulation [360]. However, apical engagement of TLR3 or TLR5 is unable to induce tolerance to subsequent basolateral TLR stimulation [360]. Nasal vaccination of OVA adjuvanted by CpG overcame the nasal tolerance and induced strong Th1 and Th2 responses through activation of TLR9 [361]. This contrasts with the responses of commensal bacteria that suppress Th17 response via TLR pathway to create an immune tolerance niche for colonization. TLR2 on CD4^+^ T cells can be activated by polysaccharide A from *Bacteroides fragilis* but not other TLR2 ligands to induce IL-10 production in the absence of APCs. Specifically, polysaccharide A treated CD4^+^Foxp3^+^ Treg cells display a more potent TLR2-dependent suppressive capacity than those treated by other TLR2 ligands [362].

The mechanism of TLR in maintaining intestinal homeostasis is not fully understood. TLR hyporesponsiveness to commensal microbiota has been suggested to play an important role in keeping homeostasis in the gut. Several mechanisms to account for this hyporesponsiveness include downregulating TLR surface expression and upregulated inhibitory Toll interacting protein with reduced phosphorylation of IRAK [363]. The hyporesponsiveness of intestinal DCs to TLR ligand engagement appears limited to TLR4 [364]. Activation of TLR3 by poly(I:C) in iECs induced retinoic acid early inducible-1 production breaks self-tolerance [365]. Thus, without commensal microbiota, the engagement of TLR in gut epithelial cells from fetal or germ-free animals can induce an inflammatory response. iECs develop TLR tolerance immediately after commensal microbial colonization [366, 367]. It has been suggested that microRNA-146a-mediated translational repression and degradation of IRAK1 are responsible for the induction of neonatal innate immune tolerance in intestinal epithelium [368]. The activation of TLR3, TLR4, TLR5 and TLR9 in iECs induces mitogen-activated protein kinase phosphatase-1 (MKP-1) mediated by NF-*κ*B signaling. MKP-1 plays an important role in the development of tolerance to TLR engagement [369]. Immunity to bacterial infection is tampered in TLR adaptor MyD88 deficient mice [370–372]. The absence of TLRs or MyD88 increased susceptibility to DSS-induced experimental colitis [360]. Administration of TLR ligands in these animals prevents the development of colitis [373]. Therefore, a base level of TLR signaling from the luminal commensal microbiota is required to maintain intestinal homeostasis [370].

A variety of DCs have been identified in intestine [374]. pDCs play an important role in the development of oral tolerance. Orally ingested antigen is presented to T cells in liver by pDCs to induce T-cell anergy or lineage deletion through a CD4^+^ T-cell-independent mechanism [375, 376]. The output of DCs from lamina propria can be increased 20–30 fold by oral administration of TLR7/8 ligand resiquimod [377]. The activation of TLR in iECs also augmented the DCs sampling of antigen through their extension into gut lumen [86]. Stimulation of human monocyte-derived macrophages with a Gram-positive commensal *Lactobacillus rhamnosus* GG or a Gram-positive pathogenic *Streptococcus pyogenes* demonstrated that both the bacteria can promote TLR2 expression in macrophages. However, only pathogenic bacteria are capable of augmenting IFN-*α*/*β*-dependent TLR3 and TLR7 gene expression. Thus, it suggested that human macrophages can discriminate the presence between commensal and pathogenic bacteria by IFN-mediated TLR gene regulation [378, 379]. Intestinal DCs also play a similar discriminative role in identification of commensal or pathogenic agents and the subsequent decision between tolerance and immunity in intestines [380].

## 5. Conclusive and Perspective Remarks

T cells play a central role in the cell-mediated immunity of the host. All subsets of T cells originate from thymocytes in thymus where they acquire their surface TCR repertoires and develop the primary phenotypic markers then migrate to peripheral lymphatic organ. Upon detection of infectious agents, T cells are activated and differentiate into effector T cells or Treg cells. TLRs are canonical members of PRRs capable of inducing T-cell activation through cross-presentation of APCs or directly acting on T cells. Activation of all the identified TLRs except TLR3 results in signaling through the MyD88-NF-*κ*B pathway. It is not known why activation of TLRs by different ligands results in different outcomes although they act via a common pathway.

The lymph node is the major peripheral lymph organ where antigen-specific responses or tolerance is triggered. As inflammation is a prerequisite to induce immune responses rather than tolerance, it is conceivable that delivery of inflammatory cytokines such as IL-12, IFN-*γ* to the tumor or its draining lymph node would be helpful to overcome the immunocompromised status in some patients, for example, in cancer patients. Thus, the immunity against cancer which has been suppressed would be reestablished in the cancer-bearing host. Indeed, intrinsic IL-12 is capable of converting Foxp3^+^ Treg cells into IFN-*γ* ^+^ Th1, IL-17^+^ Th17, or Foxp3^+^IFN-*γ* ^+^/Foxp3^+^IL-17^+^/Foxp3^+^IFN-*γ* ^+^IL-17^+^ transitional cells. The transitional Foxp3^+^IFN-*γ* ^+^ cells further differentiate into IFN-*γ* ^+^ Th1 cells but not Foxp3^+^ Treg cells although they still retain their regulatory functions at this stage [381]. Intratumoral delivery of IL-12 and granulocyte macrophage colony-stimulating factor (GM-CSF) recruits immunogenic DCs to tumors and later migrates to the local draining lymph nodes. However, these cells have a short half life and become IDO-positive tolerogenic DCs after a few days. Interestingly, the initial recruitment and activation of DCs as well as the subsequent switch to tolerogenic activity are both under the influence of IFN-*γ* [382]. It would be of interest to note whether the delivery of IL-12 to the lymph node would maintain or restore these DCs immunogenic.

Current studies support the concept of reprogramming of TLR ligands, for example, CpG ODN on Treg cells. This raises the question of whether it might be possible to overcome the immunosuppressive effects of Treg cells, for example, in patients with disordered immunity. Indeed should the Th cell be reprogrammable, the roadmap of autoimmunity therapy and/or other types of therapy would have to be reevaluated. Some disorders of immunity requiring enhanced immunosuppression can occur in the context of liver transplantation [383], kidney transplantation [384], or stem cell transplantation [385] to name a few examples. Exploiting such pathways could lead to the development of new therapeutic agents against immune disorders.

## Figures and Tables

**Figure 2:**
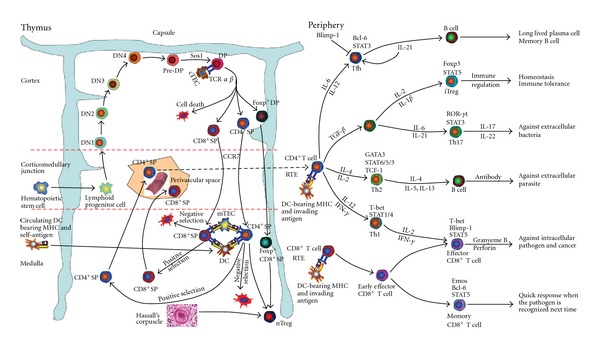
T-cell development and differentiation. It is believed that thymic lymphoid progenitor cells are derived from circulating hematopoietic stem cells originating from the bone marrow. The initial CD4/CD8 double-negative (DN) thymocytes migrate from the corticomedullary junction to the subcapsular region of the cortex and sequentially transform into DN1 (CD44^+^CD25^−^), DN2 (CD44^+^CD25^+^), DN3 (CD44^−^CD25^+^), DN4 (CD44^−^CD25^−^) and pre-DP cells, which weakly express CD4, CD8, CD25 and CD44. Then CD4/CD8 double-positive (DP) thymocytes under the influence of a guanine nucleotide exchange factor for Ras, Sos1 develop TCR*αβ* surface expression. cTECs present self-peptide-MHC complexes to TCR*αβ* to induce clonal deletion or thymocytes developing into CD4 or CD8 SP cell lineage. nTreg cell development possibly begins at the DP stage. Foxp3^+^ DP thymocytes with a functional IL-7 receptor and upregulated expression of Bcl-2 protect themselves from being negative selected. Foxp3^+^ DP thymocytes with CD103 expression are possible precursors of Foxp3^+^ CD8^+^ SP cells and finally differentiate into nTreg cells. SP cells move to the medulla through CCR7-mediated chemotaxis and interact with mTECs, which promiscuously express multifarious “tissue-specific” antigens. These antigens are taken up by DCs and cross-presented to developing thymocytes to induce negative selection establishing self-tolerance or nTreg lineage development. Circulating DCs bearing peripheral tissue antigens are also recruited intrathymically for cross-presentation. mTECs are also able to serve as APCs to induce nTreg lineage development and negative selection. Hassall's corpuscles are required to support nascent nTreg cell development. Positively selected mature thymocytes migrate through perivascular space in the corticomedullary junction and medulla and become peripheral naive T lymphocytes. When infection occurs, APCs process antigen and present epitope in combination with MHC molecules to TCR on the T-cell membrane in the presence of co-stimulatory molecules and with the help of specific cytokines to induce T-cell differentiation. IL-12 and IFN-*γ* are essential for the induction of Th1 cell. IL-4 and IL-2 are required for naive CD4^+^ T-cell differentiation into IL-4-producing Th2 cells. TGF-*β* stimulates naive CD4^+^ T cell to differentiate into Th17 cells in the presence of IL-6 or induces iTreg cell in the presence of IL-2. Th17 cells can also be induced by an alternative pathway through the cooperation of TGF-*β* and IL-21 without the participation of IL-6. Tfh cells are induced with the help of IL-6 (mice) or IL-12 (human) to produce IL-21, which backfeeds to promote Tfh cell differentiation. As a major transcription factor, T-bet along with STAT4 and STAT1 is essential for Th1 cell differentiation. Activated Th1 cell can produce IFN-*γ* and IL-2 to help CD8^+^ effector T-cell functioning. GATA3 is the Th2 master regulator. STAT6 and STAT5 are essential in Th2 cell differentiation and expansion. STAT3 cooperates with STAT6 in promoting Th2 cell development. TCF-1 participates in GATA3 activation and promotes STAT6-independent IL-4-producing Th2 cell differentiation. Th2 cells secrete IL-4, IL-5 and IL-13 to boost antibody production in B cells against extracellular parasites. Production of IL-17 by Th17 cells is ROR-*γ*t and ROR-*α* dependent. STAT3 is involved in Th17 cell differentiation, expansion and maintenance. Th17 cells participate in the immune response against extracellular bacteria by production of IL-17. Treg cell development is controlled by Foxp3 that is required for Treg lineage commitment, differentiation, expansion and function. STAT5 promotes Treg cell development by enhanced expression of Foxp3. Treg cells play a critical role in maintaining homeostasis and immune tolerance by suppression of effector cell in a cell-contact or cytokine-mediated pattern. Lineage commitment of Tfh cell is controlled by Bcl-6, while Blimp-1 plays an inhibitory effect on Tfh cell generation and function. STAT3 is necessary for Tfh cell development. Tfh cells interact with B cells in germinal center to induce generation of long-lived plasma cells and memory B cells. Naive CD8^+^ T cell primed by signals from TCR and co-stimulatory molecules differentiate into early effector cell expressing transcription factor T-bet and cytotoxic cytokines, for example, IFN-*γ*, TNF-*α* to acquire partial cytolytic abilities. The early effector cell further differentiates into late effector cell or memory cell, and this is determined by multiple factors such as the strength of IL-2R and the presence of IL-12, the presence of distinct amounts of intracellular components such as proteasome, T-bet, CD8 and IL-7R*α*, or the potency of TCR signals. T-bet and Blimp-1 are responsible for IFN-*γ* expression and participate in the cytolytic gene expression, for example, Granyeme B, Perforin to induce short-lived effector CD8^+^ T cells. STAT5 plays a critical role in maintenance of phenotype of effector CD8^+^ T cells. Eomes and Bcl-6 expressions favor memory CD8^+^ T-cells differentiation. STAT5 activation also promotes memory CD8^+^ T-cell survival.

**Figure 3:**
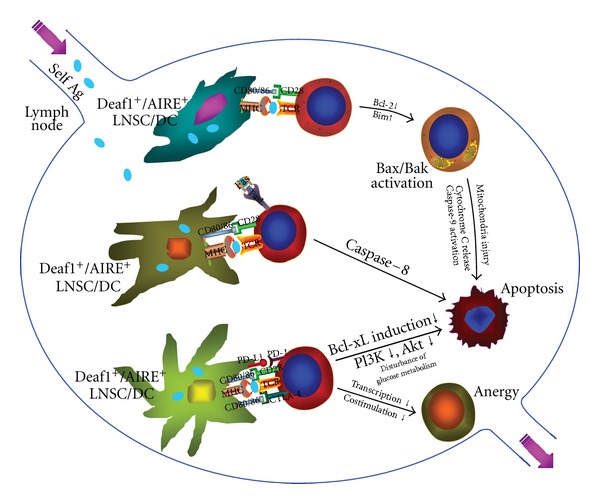
Peripheral T-cell tolerance in lymph node. All the subsets of LNSC can express PTA. AIRE and Deaf1 are involved in the regulation of this expression. Both the LNSC and follicular DC in lymph node can serve as APC to present or cross-present self-epitopes to T cells. Lymph contains abundant-processed protein fragments and peptides from draining organs or tissues and serves as a significant pool of self-antigen for the induction of peripheral tolerance. LNSC can upregulate co-stimulatory molecules to induce T-cell lineage deletion. The autoreactive T-cell lineage deletion is mediated by apoptosis mediated by Fas or Bim signals when inflammation is absent. The engagement of Fas ligand with Fas on T-cell surface triggers the apoptosis of activated T cell through caspase-dependent pathway. T-cell stimulation causes downregulation of Bcl-2 and a transient slight upregulation of Bim and this results in increased uncomplex Bim which is combined with Bcl-2 in resting status. This then activates Bcl-2 homologous antagonist/killer (Bak) and Bcl-2–associated X protein (Bax). Consequently, the integrity of mitochondria is damaged and this culminates in cell death. The tolerogenic DCs induce T-cell functional tolerance, that is, anergy by upregulation of either CTLA-4 or PD-1 expression in T cells. Augmented expression of CTLA-4 can block co-stimulatory signals by binding to CD80/86 in competition with CD28 to induce T-cell anergy. In recognition of self-antigen, PD-L1 on tolerogenic DCs interacts with PD-1 on T cells to limit T-cell activity in peripheral tissues and maintain T cell in unresponsiveness. PD-1 suppresses the PI3K induction and Akt activation. This disturbs cellular glucose metabolism and impairs T-cell survival. PD-1 activation also inhibits the cell-survival factor Bcl-xL production. CTLA-4 engagement blocks Akt phosphorylation by activation of protein phosphatase 2. Engagement of both PD-1 and CTLA-4 can significantly decrease gene transcriptions of T cell being activated.

**Table 1:** TLR expression and direct effects on T cells [268, 277, 278, 329, 333].

TLR	Location	Typical ligand	Expression in T-cell subsets			Direct effect on T cells
			Naive	Activated/Memory	iTreg	
TLR1	Cell surface	Triacryl lipopeptide	±	++	+	Increased effector T-cell proliferation and survival; abrogate the suppressive function of Treg cells
TLR2	Cell surface	Peptidoglycan	±	++	+	Increased cell proliferation and survival; promote cytotoxic activity of CTL; generate efficient memory T cells; augment Treg cell proliferation with temporal loss of suppression
TLR3	Endosome	dsRNA	+	++	−	Promote activated CD4^+^ T-cell survival
TLR4	Cell surface	Lipopolysaccharide	±	++	+	Induce Treg cell activation; enhance the suppressive function of Treg cells
TLR5	Cell surface	Flagellin	+	+	+	Augment the suppressive capacity of Treg cells
TLR6	Cell surface	Diacryl lipopeptide	+	+	+	Block the suppressive function of Treg cells
TLR7	Endosome	ssRNA	+	+	−	Augment activation/function of T cells; block the suppressive function of Treg cells
TLR8	Endosome	ssRNA	+	+	+	Augment activation/function of T cells; block the suppressive function of Treg cells
TLR9	Endosome	CpG DNA	+	++	−	Promote activated CD4^+^ T-cell survival; inhibit Treg cell suppression

++: enhanced expression; +: normal expression; ±: weak or low expression; −: expression not detectable.

## Abbreviations

AIRE: Autoimmune regulator
AP1: Activator protein 1
APC: Antigen-presenting cell
Bcl-6: B cell lymphoma 6
Bim: Bcl-2-interacting mediator of cell death
Blimp-1: B lymphocyte-induced maturation protein 1
CCR: C-C chemokine receptor
cDC: Conventional DC
CLR: C-type lectin receptor
CpG: Unmethylated cytosine preceding guanosine motif
cTEC: Cortical thymic epithelial cell
CTL: Cytotoxic T lymphocyte
CTLA-4: Cytotoxic T lymphocyte-associated antigen-4
CXCR: C-X-C chemokine receptor
MCMV: Mouse cytomegalovirus
DAP12: DNAX-activating protein of molecular mass 12 kilodaltons
DC: Dendritic cell
DD: Death domain
Deaf1: Deformed epidermal autoregulatory factor 1
DN: CD4/CD8 double-negative
DP: CD4/CD8 double-positive
dsRNA: Double-stranded RNA
DSS: Dextran sulfate sodium
Eomes: Eomesodermin
ER: Endoplasmic reticulum
FACS: Fluorescence-activated cell sorting
FADD: Fas-associated cell death domain
Foxp3: Forkhead box P3
GALT: Gut-associated lymphoid tissue
GATA transcription factors: A family of transcription factors capable of binding to the DNA sequence “GATA”
GM-CSF: Granulocyte macrophage colony-stimulating factor
HSP: Heat shock protein
IDO: Indoleamine 2, 3-dioxygenase
iEC: Intestinal epithelial cells
IFN: Interferon
IKK: Inhibitor of *κ*B kinase
IL: Interleukin
IL-12R: IL-12 receptor
IMACS: Immunomagnetic cell sorting
IRAK: IL-1R-associated kinase
IRF: Interferon regulatory factor
iTreg: Inducible regulatory T cell
I*κ*B: Inhibitor of *κ*B
ITAM: Immunoreceptor tyrosine-based activation motif
JNK: c-Jun N-terminal kinase
LAP: Latency-associated peptide
LCMV: Lymphocytic choriomeningitis virus
LEC: Lymphatic endothelial cell
LNSC: Lymph node stromal cell
LPS: Lipopolysaccharide
LRR: Leucine-rich repeat
Mal: MyD88-adapter-like
MAMP: Microbe-associated molecular patterns
MCMV: Mouse cytomegalovirus
MDA-5: Melanoma differentiation-associated gene 5
mDC: Myeloid DC
MKP-1: Mitogen-activated protein kinase phosphatase-1
mTEC: Medullary thymic epithelial cell
MyD88: Myeloid differentiation factor 88
NAK: NF-*κ*B activating kinase
NAP: NF-*κ*B activating kinase-associated protein
NEMO: NF-*κ*B essential modulator
NF-*κ*B: Nuclear factor *κ*B
NLR: Nucleotide binding domain and leucine-rich repeat containing gene family
nTreg: Natural regulatory T cell
ODN: Oligodeoxynucleotide
OVA: Ovalbumin
PAMP: Pathogen-associated molecular patterns
PD-1: Programmed death 1
pDC: Plasmacytoid DC
PI3K: Phosphatidylinositol 3-kinase
poly(I:C): Polyinosinic-polycytidylic acid
PRR: Pattern recognition receptor
PTA: Peripheral tissue-restricted antigen
RHIM: Receptor-interacting protein homotypic interaction motif
RIG-I: Retinoic acid-inducible gene-I
RIP: Receptor-interacting protein
RLR: Retinoic acid-inducible gene-I-like receptor
ROR: Retinoic acid receptor related orphan receptor
RTE: Recent thymic emigrants
SCID: Severe combined immunodeficiency
Sos1: Son of sevenless gene 1
SP: CD4/CD8 single-positive
ssRNA: Single-stranded RNA
STAT: Signal transducer and activator of transcription
TAB: Transforming growth factor *β*-activated kinase-1 binding protein
TAK: Transforming growth factor *β*-activated kinase
T-bet: T box expressed in T cell
TBK: TRAF family member-associated NF-*κ*B activator-binding kinase
TCF-1: T-cell factor 1
Tcm: Central memory T cell
TCR: T-cell receptor
Tem: Effector memory T cells
Tfh: T follicular helper cell
TGF: Transforming growth factor
Th: T helper cell
TICAM-1: TIR domain containing adaptor molecule-1
TIR: Toll/IL-1 receptor domain
TIRAP: TIR domain-containing adapter protein
TLR: Toll-like receptor
TNF: Tumor necrosis factor
TRAF: Tumor necrosis factor receptor associated factor
TRAM: TRIF-related adaptor molecule
TREM-2: Triggering receptor expressed on myeloid cell-2
TRIF: TIR domain-containing adaptor inducing interferon-*β*
TSA: “Tissue-specific” antigen
Tscm: Memory stem T cells.
